# Complex vectorial optics through gradient index lens cascades

**DOI:** 10.1038/s41467-019-12286-3

**Published:** 2019-09-19

**Authors:** Chao He, Jintao Chang, Qi Hu, Jingyu Wang, Jacopo Antonello, Honghui He, Shaoxiong Liu, Jianyu Lin, Ben Dai, Daniel S. Elson, Peng Xi, Hui Ma, Martin J. Booth

**Affiliations:** 1 Department of Engineering Science, University of Oxford, Parks Road, Oxford, OX1 3PJ UK; 2 Department of Physics, Tsinghua University, 100084 Beijing, China; 3 Shenzhen Key Laboratory for Minimal Invasive Medical Technologies, Institute of Optical Imaging and Sensing, Graduate School at Shenzhen, Tsinghua University, 518055 Shenzhen, China; 4 Shenzhen Sixth People’s Hospital (Nanshan Hospital) Huazhong University of Science and Technology Union Shenzhen Hospital, 518052 Shenzhen, China; 5 Hamlyn Centre for Robotic Surgery, Institute of Global Health Innovation, Imperial College London, London, SW7 2AZ UK; 6 School of Data Science, City University of Hong Kong, Kowloon, Hong Kong China; 7 Department of Biomedical Engineering, College of Engineering, Peking University, 100871 Beijing, China

**Keywords:** Applied optics, Optical techniques, Imaging techniques

## Abstract

Graded index (GRIN) lenses are commonly used for compact imaging systems. It is not widely appreciated that the ion-exchange process that creates the rotationally symmetric GRIN lens index profile also causes a symmetric birefringence variation. This property is usually considered a nuisance, such that manufacturing processes are optimized to keep it to a minimum. Here, rather than avoiding this birefringence, we understand and harness it by using GRIN lenses in cascade with other optical components to enable extra functionality in commonplace GRIN lens systems. We show how birefringence in the GRIN cascades can generate vector vortex beams and foci, and how it can be used advantageously to improve axial resolution. Through using the birefringence for analysis, we show that the GRIN cascades form the basis of a new single-shot Müller matrix polarimeter with potential for endoscopic label-free cancer diagnostics. The versatility of these cascades opens up new technological directions.

## Abstract (web summary)

The manufacturing process for GRIN lenses causes a symmetric birefringence variation which is considered a deficiency. Here, the authors show how this birefringence can generate vector vortex beams and form the basis of a Müller matrix polarimeter with potential for endoscopic label-free cancer diagnostics.

## Introduction

Graded index (GRIN) lenses have a varying refractive index profile that enables focusing or imaging through a compact rod-like structure^1–8^. The lower mass and size of GRIN lenses mean that are preferable to other conventional optics in many applications: shorter ones can be used as coupling lenses in fibre systems or in waveguide-based on-chip devices;^1,2,6^ longer counterparts are normally treated as relay lenses or objective lenses that can form biopsy needles for disease diagnosis. They can also be found in other compact systems, such as fluorescence micro-endoscopes for application in deep tissue imaging^1,4,8^.

**Fig. 1:**
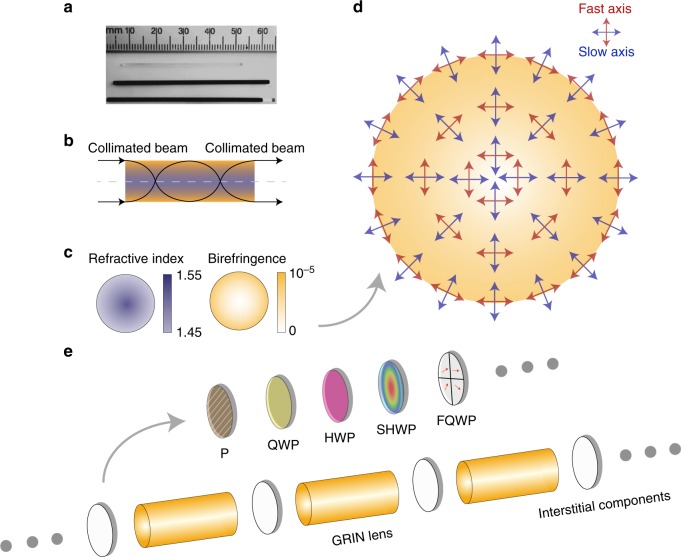
GRIN lens properties and GRIN lens cascade. **a** Commercial GRIN lenses. **b** Schematic of the GRIN lens ray trace. **c** The birefringence and refractive index profile of a GRIN lens over its cross-section. **d** The fast axis/slow axis distribution of the local retardation across the GRIN lens. **e** The GRIN lens cascade. This cascade consists of the combination of one or more GRIN lenses along with various devices including interstitial components—such as P: polarizer; QWP: quarter waveplate; HWP: half waveplate; SHWP: spatially variant half waveplate; FQWP: four quadrant quarter waveplate array with four different fast axis orientations, and so on

Procedures for fabrication of GRIN lenses are well established: an ion-exchange process creates a rotationally symmetric index profile in the glass rod. There is however an undesirable side effect: the process also introduces a concomitant intrinsic birefringence that maintains the same rotational symmetry^9,10^ (see Fig. 1a–c). This gradually changing birefringence profile exhibits the following properties: (1) the magnitude of the retardance is constant at a given radius, (2) the retardance increases with increasing radius; and (3) the slow axis is oriented in the radial direction. These properties mean that the GRIN lens behaves like a spatially variant waveplate array, providing a continuum of birefringence states that can manipulate the polarization and phase of a light beam (see Fig. 1d and details in Supplementary Note 1).

Here, through better understanding of these phenomena, we have drawn upon these previously undesirable GRIN lens properties to build new light manipulation structures to extend the applications of traditional GRIN lens systems (GLSs). We call these structures “GRIN lens cascades.” As illustrated in Fig. 1e, these cascades comprise one or more GRIN lenses with interstitial optical elements, including polarizers and waveplates. Such combinations of elements introduce myriad possibilities for structuring the phase and polarization profiles of beams and foci. We describe here several aspects that enable novel extra functionality in GLSs. We first show that GRIN lenses (and, furthermore, GRIN cascades) can generate a multitude of vector vortex beams (VVBs), and permit the creation of corresponding vector foci. We also demonstrate that, by proper choice of the interstitial optics of the cascade, we can use azimuthal or radial polarization light fields at the GRIN lens input to enhance the axial resolution. Lastly, we show how a GRIN cascade forms the basis of a novel single-shot Müller matrix (MM) polarimeter, which can comprehensively extract the polarization characteristics of an object. This capability can facilitate GLS-based label-free endoscopic diagnosis of structural changes in tissue, with clear applications to cancer detection.

## Results

### Vectorial beam generation by GRIN lens cascades

Great interest has developed in vector light fields that contain complex phase and polarization structures, whether in the focal or pupil domains. Of special interests are those containing singularities, as they promise unprecedented capabilities for applications ranging from classical to quantum optics^11–17^. Examples include various complex vector beams that have non-uniform polarization distribution across the transverse plane^18–22^ or beams with helicoidal wave fronts that carry orbital angular momentum (OAM)^23–31^, or indeed their combinations as VVBs^32,33^. Currently, such beams can be generated by using a range of modulation devices, such as spatial light modulators, q-plates^34^, metasurfaces^35^, segmented waveplates^36^, or Fresnel-reflected cones^33^. We present here further options enabled by the birefringence of GRIN lenses, especially for their usage in GLSs. The focusing properties of the GRIN lens arise due to the refractive index-led dynamic phase that accounts for the sinusoidal ray trace using scalar theory. However, the birefringence means that vector effects must be considered. Furthermore, the lens is capable of manipulating the light through the introduction of a geometric Pancharatnam–Berry phase (PB phase)^37^. To the best of our knowledge, this is the first time that the PB phase and its corresponding effects have been considered for GLSs.

**Fig. 2:**
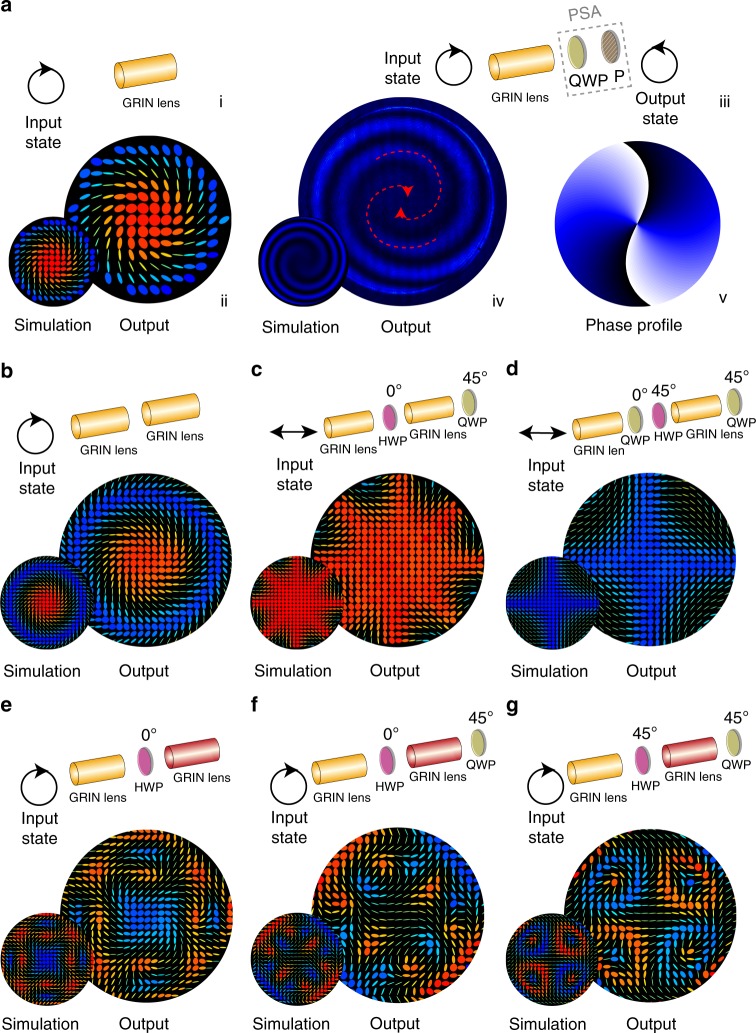
VVBs generation through GRIN lens cascades. **a** (i) Schematic of a single GRIN lens cascade with (ii) right-hand circular input, output polarization fields for both simulation and experimental results (the red side of the colour scale indicates right-hand circular, whereas blue corresponds to left-hand circular). Note that before the GRIN lens cascade, there is a polarization state generator (PSG) to generate a chosen arbitrary polarization state; following the GRIN lens is a polarization state analyser (PSA) that enables Stokes vector measurement (see methods in Supplementary Note 3). Except in (iii) the PSG and PSA parts are omitted for clarity. (iii) An illustration for schematic in **a** with right-hand circular input and left hand circular analysis. The two spirals in the interferogram (iv) and the phase profile (v) indicated that the light beam contained two units of OAM. **b**–**g** show higher-order GRIN lens cascades and their generated light fields under specific inputs. In **e**–**g** the red-coloured GRIN lens indicate another GRIN type with a larger magnitude retardance profile almost equivalent to a sequence of three of the first (orange) type of lens (for details see Supplementary Note 4)

We first demonstrated VVB generation ability by using a single GRIN lens cascade. Examples of beam generation are shown in Fig. 2a through simulation and experiment, which show close correspondence (see methods in Supplementary Notes 2, 4, and 6). It can be found from the polarization pattern that the beam is a specific singular vector beam, known as a full Poincaré beam^38,39^, that contains polarization singularities (see mathematical validation and polarization singular analysis in Supplementary Note 4). An interesting consequence of this is that the beam contains components of OAM, which have arisen due to the PB phase (see theory in Supplementary Note 6). This was verified using a polarization state analyser (PSA) to select the left-hand circular polarized state, and followed the commonly used practice of using interference patterns^33,35^ (see methods in Supplementary Note 5); the existence of two spirals validated that the single GRIN lens cascade could generate two units of OAM (see Fig. 2a (iii)–(v)).

This concept can be extended further. Higher-order GRIN lens cascades—those containing more than one GRIN lens, which may or may not be the same—are able to create even more complex VVBs, as shown in Fig. 2b–g. These results reveal that higher-order cascades can generate complex polarization fields that have variations in radial or azimuthal directions, or encompass multiple sub-beams, each of which constitutes a full Poincaré beam; each of these Poincaré beams could have different topological charge or handedness, as well as beam profile (see analysis in Supplementary Note 4). The beams also contain different complex phase profiles; in Supplementary Note 6 we illustrated further this phase manipulation ability.

It is important to realize that the spatial modulation effects described here are not confined to controlling the beam profile for the corresponding GLSs. They also have applications in focal shaping (as follows in section “Point spread function modulation” and Discussion), such as in fluorescence-based GLS micro-endoscope, and application in GLS-based polarization detection (as explained in section “Vectorial beam analysis” and Discussion), which could enable label-free sensing.

### Point spread function modulation by GRIN lens cascades

Due to the inherent link between the beam profile and the focus, polarization and/or phase modulation via the cascade also affects the point spread function (PSF) of a GLS. As beam modulations can also be harnessed in many imaging methods to enhance spatial resolution or other imaging properties^19,20,40^, GRIN lens cascades have expected benefits in various GLSs. Indeed, we can show that through understanding these previously unwanted birefringence phenomena, it is possible to improve the performance of current GLSs, specifically as used for scanning micro-endoscopy.

Previous research has shown that GLS used in biomedical imaging can suffer from a lower than expected axial resolution; normally this is attributed to spherical aberration^41–43^. This has been a particular problem when using multiple pitch or long GRIN lenses, where the PSF has shown significant elongation^8^. We show here that this problematic phenomenon can be also introduced by the inherent birefringence of the GRIN lens (via the induced spatial polarization aberration). Furthermore, through understanding of these phenomena, we present a possible solution based upon specific polarization light fields that improves the axial resolution.

The fast and slow axis distributions of the GRIN lens (Fig. 1d) imply that the GRIN lens has two polarization eigenmodes, for which the beams would maintain the same polarization profiles throughout the device. These two modes—which are azimuthal and radial linearly polarized light fields—experience their own refractive index profiles *n*_o_(*r*) and *n*_e_(*r*), which are still approximately quadratic with *r*, but have different magnitudes. Hence, their focusing strength is different and the corresponding focusing pitch within the GRIN lens is different. As a consequence, the positions of the two corresponding foci are axially shifted relative to each other in the focal region (for the detailed mechanism see Supplementary Note 2). For this reason, other input states that are a combination of these eigenmodes—including uniform polarization states—create a superposition of both axially offset foci that lead to the elongated focus. However, this problem can be avoided if a single eigenmode is used.

**Fig. 3:**
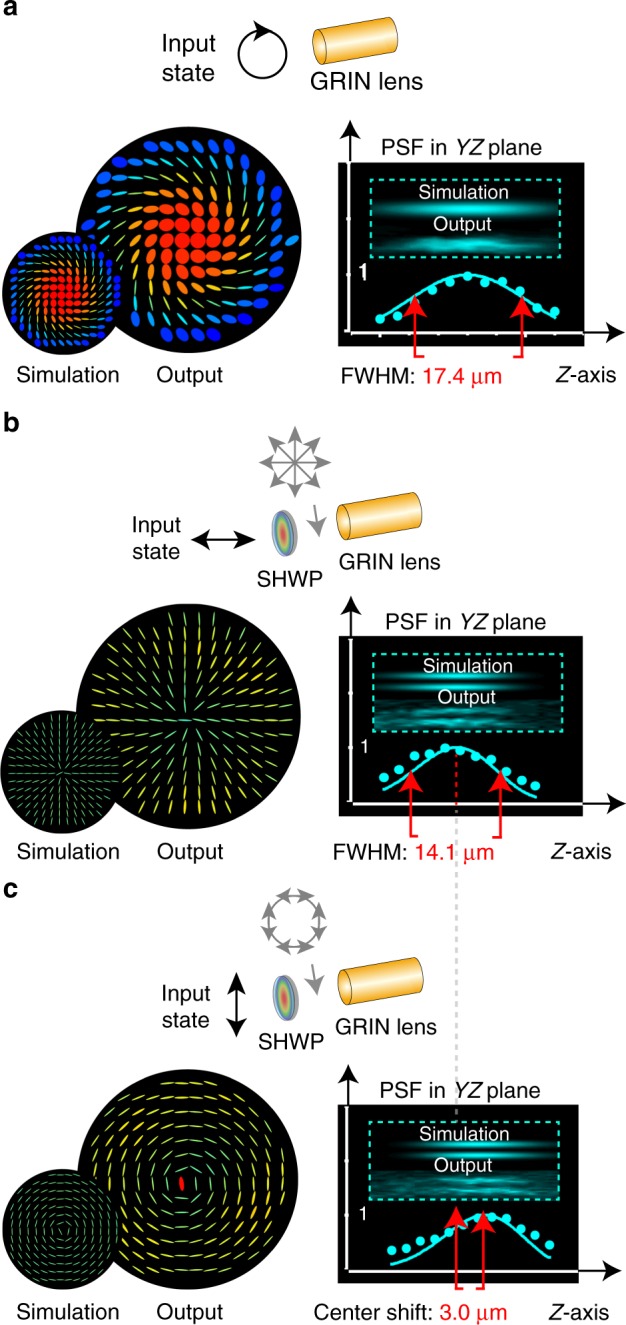
PSF modulation through GRIN lens cascade. **a** Schematic of a single GRIN lens cascade illuminated by right-hand circular polarized light, beam profile after the GRIN lens, and the corresponding axial PSF including the FWHM. **b**, **c** show the GRIN lens cascade including SHWP with horizontal and vertical linear polarized light input, beam profile after the GRIN lens, and the corresponding axial PSF to demonstrate the focus shift when using the radial/azimuthal polarization input light fields

To verify this proposition experimentally, we first measured the focus of GRIN lens when the input was a mixture of the two eigenmodes, using uniform circular polarization at the input (Fig. 3a). The focal spot was elongated along the axis, with full-width at half-maximum (FWHM) of 17.4 μm (see Supplementary Note 2 for the details of GRIN lens and methods). The inclusion of a spatially variant half waveplate (SHWP) into the cascade, combined with appropriate input polarization, permitted generation of the two eigenmodes. The pupil fields at the output, as well as the axial PSF, were measured. We can see in Fig. 3b that the FWHM decreased to 14.1 μm and, in Fig. 3c, that a focal shift of 3.0 μm was present between the foci of the two eigenmodes, confirming our theory. Note that the foci for the individual eigenmodes were ring shaped in the lateral plane, with zero intensity along the axis due to the phase singularity introduced by the SHWP in the cascade. In a practical imaging system, this effect could be compensated using an appropriate helicoidal phase plate^44^, or an alternative method for preparation of the input polarization state. Our observations show that enhanced axial resolution can be obtained in a GLS by modulation of the focus through the appropriate configuration of the input polarization and phase state.

### Vectorial beam analysis through GRIN lens cascades

As shown in the previous section, there is a complex interdependence of the focal field and the polarization state of the input beam, as processed through the GRIN lens cascade. This dependence provides further opportunities to harness the birefringence properties of the GRIN lens as parallel analysis channels for polarization sensing. The primary ability of the GRIN lens as a focusing device is due to its continuously varying, rotationally symmetric refractive index distribution^9,10^. As already discussed, this is accompanied by the gradually varying birefringence distribution. As a high degree of symmetry is required for focusing, this means that the birefringence distribution is also highly symmetric. This provides a continuous, non-pixellated, range of analysis channels that could be used for polarimetry.

MM polarimetry is a commonly used method to measure the full polarization properties of materials, particularly in biomedical applications, where its ability to characterize tissue structures in a label-free manner is highly advantageous^45–55^. Numerous MM polarimeters have been proposed, many of which are based on sequential generation of states of polarization (SOP) and analysis by rotation of polarization components or modulation of variable retarders^48^. However, in general, sequential measurement is not suitable for fast detection, such as for moving objects where it leads to unexpected measurement errors^51^. The speed can also limit the application of MM polarimetry to in vivo detection of a dynamic sample, such as in clinical applications.

**Fig. 4:**
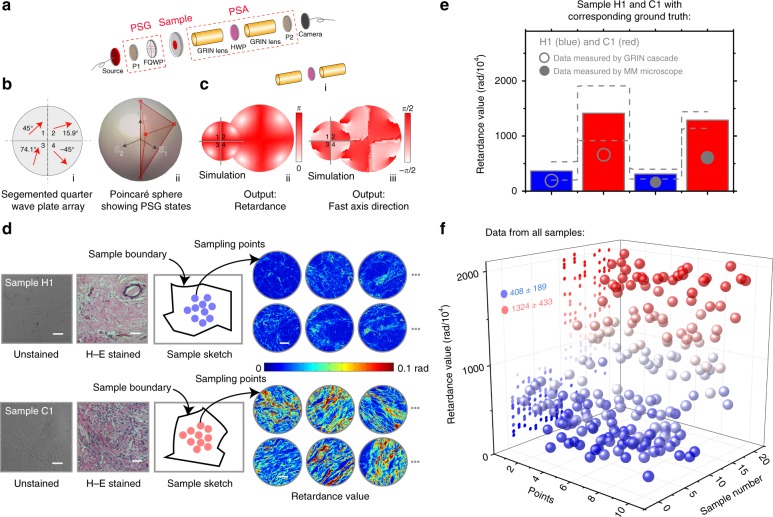
Characterization of the GRIN lens-based MM polarimeter and measurement of tissue samples. **a** Simplified setup of the polarimeter. PSG: P1, polarizer; FQWP, four-quadrant quarter waveplate, with different fast axis orientations in each quadrant. PSA: P2, polarizer; HWP, half waveplate. **b** (i) and (ii) details of FQWP and the Poincaré sphere showing the SOPs generated by the PSG. **c** (i) The GRIN lens cascade in the PSA besides the P2. (ii) and (iii) Simulation and experimental results of retardance and the fast axis orientation of (i), derived by the MMPD method. **d** Demonstration of the samples H1 and C1 (unstained samples and their H–E-stained counterparts), sketches with corresponding random sampling points (see methods in Supplementary Note 10) and examples of retardance distributions measured by the MM microscope (as ground truth). Scale bar: 50 μm. **e** Statistic histogram (mean value and the standard deviation of the retardance) of the 10 points from each sample within the same region, measured by the GRIN lens cascade and a conventional MM microscope. Standard deviations are shown via the dashed lines. Numbers refer to Supplementary Table 1. Source data are provided as a Source Data file. **f** Overall data distribution (retardance value). The overall number of samples was 20 (half cancerous vs. half healthy, giving 10 testing points per sample). Note that blue (ball or histogram) represents healthy samples, while red represents cancerous samples

We propose here a new compact single-shot MM polarimeter based on a GLS—specifically, a dual GRIN lens cascade—which can directly act as a basis of rigid endoscopic system. It includes a PSG consisting of a polarizer and a four-quadrant quarter waveplate (Fig. 4b (i)) to generate simultaneously four different illumination SOPs. Figure 4b (ii) shows the optimized SOPs on the Poincaré sphere as generated by the PSG (see design details in Supplementary Note 7). The GRIN lens cascade-based PSA is shown in Fig. 4a; here, a half-waveplate was sandwiched between two identical GRIN lenses, which were followed by a polarizer. We applied the Lu–Chipman MM polar decomposition (MMPD)^56^ method (see methods in Supplementary Note 1) to assess the equivalent retardance and fast axis direction of the PSA—these are shown for both simulation and experiment in Fig. 4c (ii) and (iii). Each of the four quadrants of the PSA provides access to a continuum of polarization analysis channels. Taking advantage of these channels, the GRIN lens cascade can be employed as a complete analyser to calculate four Stokes vectors in a single shot—one for each of the four channels, which thus enables the single-shot MM measurement (see details of characterization in Supplementary Notes 8 and 9).

To illustrate the potential of the GLS polarimetry for clinical diagnosis, the system was tested on tissue samples. We measured the MMs of the unstained 12-μm-thick human breast ductal carcinoma tissue samples at two stages (healthy vs. invasive carcinoma) and we derived the retardance parameter from the MMs to permit quantitative comparison (see methods in Supplementary Note 1). Clinically, different stages of breast ductal carcinoma tissues have different proportions and distribution of fibrous structures in and around the milk ducts^52^. An important potential application of MM polarimetry is to distinguish between such stages of cancerous tissue in a label-free manner^52^. We first demonstrated the method on one healthy tissue sample (named sample H1, where H represents healthy) and one carcinoma tissue sample (named sample C1, where C represents cancerous). The number of the random sampled points measured by the GRIN lens cascade for each was fixed at 10 throughout this work (see Fig. 4d, sample information and corresponding methods in Supplementary Note 10). Figure 4e, f reveal the statistical distribution of data measured by the cascade and MM microscope, as ground truth (see data details and the corresponding *P* value analysis in Supplementary Note 10 and Supplementary Table 1). We found from the data that the measured values of retardance for sample C1 were significantly larger than those of the sample H1 just as the same significant difference existed among the data measured by MM microscope. Furthermore, the measured values from all 10 breast ductal carcinoma samples (1324 ± 433 rad/10^4^) were significantly larger than those of the 10 healthy ones (408 ± 189 rad/10^4^)—these retardance measurements were calculated from the original measured data (in Fig. 4f) through standard statistical methods^52^ to obtain their mean values and standard deviations (see details in Supplementary Note 10). The difference is due to the larger proportion of fibrous structures present in the cancerous tissues, which is a consequence of the inflammatory reactions induced by cancer cells^52^. The results reveal that the GRIN lens cascade-based polarimeter has the ability to differentiate between healthy and cancerous breast tissues.

These experiments show that the GRIN cascades can take advantage of the previously unwanted birefringence not only to create vector foci and improve axial resolution in the focus but also to enable polarimetric measurement. This has potential to act as a single-shot, compact, polarization detection probe to assist minimally invasive clinical diagnosis.

## Discussion

By understanding and harnessing the inherent—and previously undesirable—birefringence of GRIN lenses, we have opened up new possibilities for their use. Combining one or more GRIN lenses into cascades with other optical elements provides a wide range of complex beam modulation capabilities to benefit current GLSs, including VVB generation, focal spot shaping, and in polarization analysis. All of those demonstrations are achieved using standard, off-the-shelf optical components and compatible with the GRIN lens’ basic functions as a focusing and imaging device.

The fundamental realizations here about the joint effects of the intrinsic birefringence and the focusing properties of the GRIN lens are also important in understanding some of the current challenges in use of various GLSs^1–8^. Our observations on the dimensions of the pupil/focal distributions, the use of polarization eigenmodes, and the improvement of axial resolution show that better understanding of their vector modulation effects can benefit current GLSs, such as mitigation against aberration problems in GRIN lens-based two-photon fluorescence micro-endoscopy. Furthermore, the well-controlled creation of ring-shaped foci through vectorial beam manipulation could now enable further lateral resolution enhancements for GLS through stimulated emission depletion^19,20^, MINFLUX^57^ or related microscopy methods. These methods are particularly sensitive to polarization and phase aberrations, so improved understanding of the birefringence effects and their mitigation will be essential if they are to be combined with GLSs. This may find application in in vivo deep tissue (such as brain) imaging, which cannot be easily achieved by alternative objective lenses. Combination of the GRIN cascades with active optical elements^58^ in cascade, such as a spatial light modulators, could enable further applications in beam or focal control. Further imaging methods for GLSs are then expected across various existing techniques such as structured illumination microscopy^59^.

Our observations that the GRIN cascades perform optically efficient, continuous, non-pixellated, vectorial beam modulation have important consequences for many applications. The geometric phase^16^ effects generated within the GRIN cascades are more complex than those from planar waveplate arrays, not only in the spatial domain but also in the time domain (see details in Supplementary Note 2), which may open up interesting prospects for quantum state manipulation. The observation of OAM generation could also have potential for further GRIN lens-based micro-manipulation applications, which take advantage of the optical torques introduced by the phase vortex. As certain cascades show highly asymmetric MMs (see details in Supplementary Note 1), there is scope for further investigation of these unusual properties. The same cascades also exhibit gradients in circular anisotropy, which presents the intriguing possibility that the spin-Hall effect of light could be harnessed in a GRIN lens-based imaging system (see details in Supplementary Note 1). Perhaps, the most immediate application of this new concept is in polarimetry. The GRIN cascade provides the basis a miniaturizable, cost-effective, compact, and multifunctional probe with the capability for (endoscopic^55^) MM polarimetric diagnosis in the clinical setting and it also has the potential for widefield or scanned imaging. The demonstrations in this article were performed at a single wavelength, but could be expanded to multiple wavelength channel polarimetry, while still maintaining optimal MM measurement for each wavelength.

Overall, the optical properties of GRIN lenses and their combination in cascade structures provide a wealth of opportunity for further development. Future benefits are expected in a wide range of applications, spanning from quantum optics to clinical diagnosis.

### Reporting summary

Further information on research design is available in the Nature Research Reporting Summary linked to this article.

## Supplementary information

Supplementary Information

Reporting Summary

Source Data

## Data Availability

Data generated and analysed during this study are included in this article and its Supplementary Information files, and are also available from the corresponding authors on reasonable request. The source data underlying Fig. 4e and Supplementary Fig. 11c are provided as a Source Data file.
