# Supplementary material for: Complex vectorial optics through gradient index lens cascades
Source: Nat Commun. 2019 Sep 19;10:4264. doi: 10.1038/s41467-019-12286-3 (PMC6753074; doi:10.1038/s41467-019-12286-3)
Supplement: Supplementary file 1 — Supplementary Information [file 41467_2019_12286_MOESM1_ESM.pdf]

Complex vectorial optics through gradient index lens cascades

– Supplementary Information –

He et al.

Supplementary Notes

Chao He[1,*], Jintao Chang[2,3], Qi Hu[1], Jingyu Wang[1], Jacopo Antonello[1], Honghui He[3], Shaoxiong Liu[4], Jianyu Lin[5], Ben Dai[6], Daniel S. Elson[5], Peng Xi[7], Hui Ma[2,3] and Martin J. Booth[1,*]

[1]*Department of Engineering Science, University of Oxford, Parks Road, Oxford, OX1 3PJ, UK*

[2]*Department of Physics, Tsinghua University, Beijing 100084, China*

[3]*Shenzhen Key Laboratory for Minimal Invasive Medical Technologies, Institute of Optical Imaging and Sensing, Graduate School at Shenzhen, Tsinghua University, Shenzhen 518055, China*

[4]*Shenzhen Sixth People's Hospital (Nanshan Hospital) Huazhong University of Science and Technology Union Shenzhen Hospital, Shenzhen 518052, China*

[5]*Hamlyn Centre for Robotic Surgery, Institute of Global Health Innovation, Imperial College London, London SW7 2AZ, UK*

[6]*School of Data Science, City University of Hong Kong, Kowloon, Hong Kong, China*

[7]*Department of Biomedical Engineering, College of Engineering, Peking University, Beijing 100871, China*

[*]*Corresponding authors: chao.he@eng.ox.ac.uk; martin.booth@eng.ox.ac.uk*

Supplementary Note 1: Characterization of polarization properties of the GRIN lens

The polarization properties of an object can be described using either a Jones matrix (JM) or Müller matrix (MM)[1, 2]. While Jones matrices are used to deal with interference cases by fully polarized light, the MM contains 16 elements m_{kl} (k, l = 1,2,3,4), which can comprehensively represent the polarization characteristics – including depolarization – of the target[1, 2]. In this section, we use the MM to characterize the polarization properties of GRIN lens cascades. These measurements use a calibrated MM imaging polarimeter based upon the dual rotating waveplates method[3-5]. The measurement principle and schematic of the setup are shown in the following contexts.

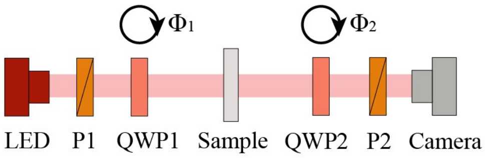

Supplementary Figure 1. Setup of the MM imaging polarimetry. LED light source; P1, P2: fixed polarizer; QWP1, QWP2: rotated quarter waveplate. Camera as detector.

Supplementary Figure 1 shows the MM imaging polarimeter. The polarizers (P1, P2) are oriented in the same direction. Two quarter waveplates (QWP1, QWP2) rotate with fixed rotational speeds, such that $\phi_1 = 5\phi_2$. The main measurement principle is shown in Eq. (1) below, where superscript q represents the qth measurement. M_{Sam} is the MM of the sample, M_{P1}, M_{P2}, M_{QWP1}, M_{QWP2} are MMs of P1, P2, QWP1, QWP2, respectively. M_{System} is the equivalent overall MM of the system. S_{in} and S_{out} are incident and output Stokes vectors.

$$S_{\text{out}}^q = M_{\text{System}} S_{\text{in}} = M_{\text{P2}} M_{\text{QWP2}} M_{\text{QWP2}}^q M_{\text{Sam}} \quad M_{\text{QWP1}}^q M_{\text{P1}} S_{\text{in}}. \tag{1}$$

Since the intensity is equivalent to the first element S_0 of the Stokes vector, we make $I^q = (S_{\text{out}}^q)_0$, which represents the corresponding intensity of qth measurement. From Eq. (1) we obtain the Fourier series,

$$I^q = (S_{\text{out}}^q)_0 = a_0 + \sum_{n=1}^{12} (a_n \cos 2n\phi_1^q + b_n \sin 2n\phi_1^q), \tag{2}$$

where a_n and b_n are the Fourier coefficients, and ϕ_1^q is the angle of QWP1 at q^{th} measurement. The MM of the sample could then be calculated from the Fourier coefficients as shown below. For more details see Ref [2-4].

$$M_{\text{Sample}} = \begin{pmatrix} m_{11} & m_{12} & m_{13} & m_{14} \\ m_{21} & m_{22} & m_{23} & m_{24} \\ m_{31} & m_{32} & m_{33} & m_{34} \\ m_{41} & m_{42} & m_{43} & m_{44} \end{pmatrix} =$$

$$\begin{pmatrix} a_0 - a_2 + a_8 - a_{10} + a_{12} & 2a_2 - 2a_8 - 2a_{12} & 2b_2 - 2b_8 - 2b_{12} & b_1 - b_9 - b_{11} \\ -2a_8 + 2a_{10} - 2a_{12} & 4a_8 + 4a_{12} & 4b_{12} - 4b_8 & -2b_9 + 2b_{11} \\ -2b_8 + 2b_{10} - 2b_{12} & 4a_8 + 4b_{12} & 4a_8 - 4a_{12} & 2a_9 - 2a_{11} \\ b_3 - b_5 + b_7 & -2b_3 - 2b_7 & -2a_3 + 2a_7 & -a_4 + a_6 \end{pmatrix}. \quad (3)$$

Once the MM of the sample is obtained, we apply the Lu-Chipman MM polar decomposition method (MMPD)[6] to extract polarization parameters from the MM. This method is widely used to decompose the complicated interactions between sample and polarized light into a series of phenomena[6-10]: the sample's diattenuation (D), depolarization (Δ), retardance (R) and its fast axis direction (θ) represented by the corresponding three Matrix factors M_Δ, M_R, M_D, respectively. The main principle is represented by Eq. (4).

$$M_{\text{Sam}} = M_\Delta M_R M_D. \quad (4)$$

The diattenuation value D can be readily obtained from the second to fourth elements in the first row of a MM, that is the elements m_{12}, m_{13} and m_{14} respectively, as shown in Eq. (5). The retardance R is reconstructed from the trace of M_R, Eq. (6), while the orientation of optic axis ranging from $-\frac{\pi}{2}$ to $\frac{\pi}{2}$ radians is calculated according to Eq. (7). The depolarization properties are included in the in the bottom right 3×3 matrix m_Δ of the matrix M_Δ, which is shown in Eq. (8). λ_1, λ_2 and λ_3 in Eq. (8) are the eigenvalues of m_Δ, and P is a matrix composed of the eigenvectors of m_Δ. Δ_L and Δ_c in Eq. (8) are linear/circular depolarization values.

$$D = \sqrt{m_{12}^2 + m_{13}^2 + m_{14}^2}, \quad (5)$$

$$R = \cos^{-1}\left[\frac{\text{tr}(M_R)}{2} - 1\right], \quad (6)$$

$$\theta = \frac{1}{2}\tan^{-1}\left[\frac{M_{R23}-M_{R32}}{M_{R3}\ -M_{R13}}\right], \tag{7}$$

$$m_{\Delta} = P\begin{pmatrix}\lambda_1 & 0 & 0\\ 0 & \lambda_2 & 0\\ 0 & 0 & \lambda_3\end{pmatrix}P^{-1},\ \ \Delta_L = 1-\frac{\lambda_1+\lambda_2}{2},\ \ \Delta_R = 1-\lambda_3. \tag{8}$$

Supplementary Figures 2a to 2c demonstrate the MMs and the corresponding MMPD results of the single GRIN lens cascade. We found that 1) the measured experimental MM as well as the MMPD parameters matched well with the simulation counterparts; 2) the MMPD results reveal that the birefringence structure of the GRIN lens behaves equivalently to a spatially-variant waveplate array with a) linear retardance that gradually increases along the radial direction and b) fast axis directions that vary azimuthally (gradually changing from -π/2 to 0 then to π/2 radians as the azimuthal angle changes from 0 to π radians, then a similar variation when the azimuthal angle changes from π to 2π radians). The fast/slow axis direction distributions are shown in Fig. 1b in the main article as well.

In Supplementary Figures 2d to 2g, we further show four different SHWP based GRIN lens cascades including in Supplementary Figure 2d – the one used in the main article (as Fig. 3b and 3c). Besides the close correspondence between simulation and experimental results, we also found that the MMs of these GRIN lens cascades have significant off-diagonal asymmetry which is of interest in MM research, as well as related sample information analysis[11]. Specifically, we extracted the circular anisotropy coefficient[12] of these four MMs, shown in Supplementary Figure 2h, in which we use grey arrows to indicate the gradient of the circular anisotropy[13]. This implies the system can be used to demonstrate spin-Hall effect (SHE) of light[13]. Both of these phenomena deserve further exploration.

We propose here that the GRIN lens cascade can also find use as a special device for other research areas, such as the derivation of quantitative MM parameters from exotic materials (e.g. chiral characteristics[14]), biomedical research (multi-layer birefringence and/or diattenuation characterization[11]), or studies on complex spin-orbital interaction (SOI) processes of light[13]. These all take advantage of the unique property of the GRIN lens – its equivalence to a spatially variant waveplate array, which can encompass all combinations of retardance and fast axis direction.

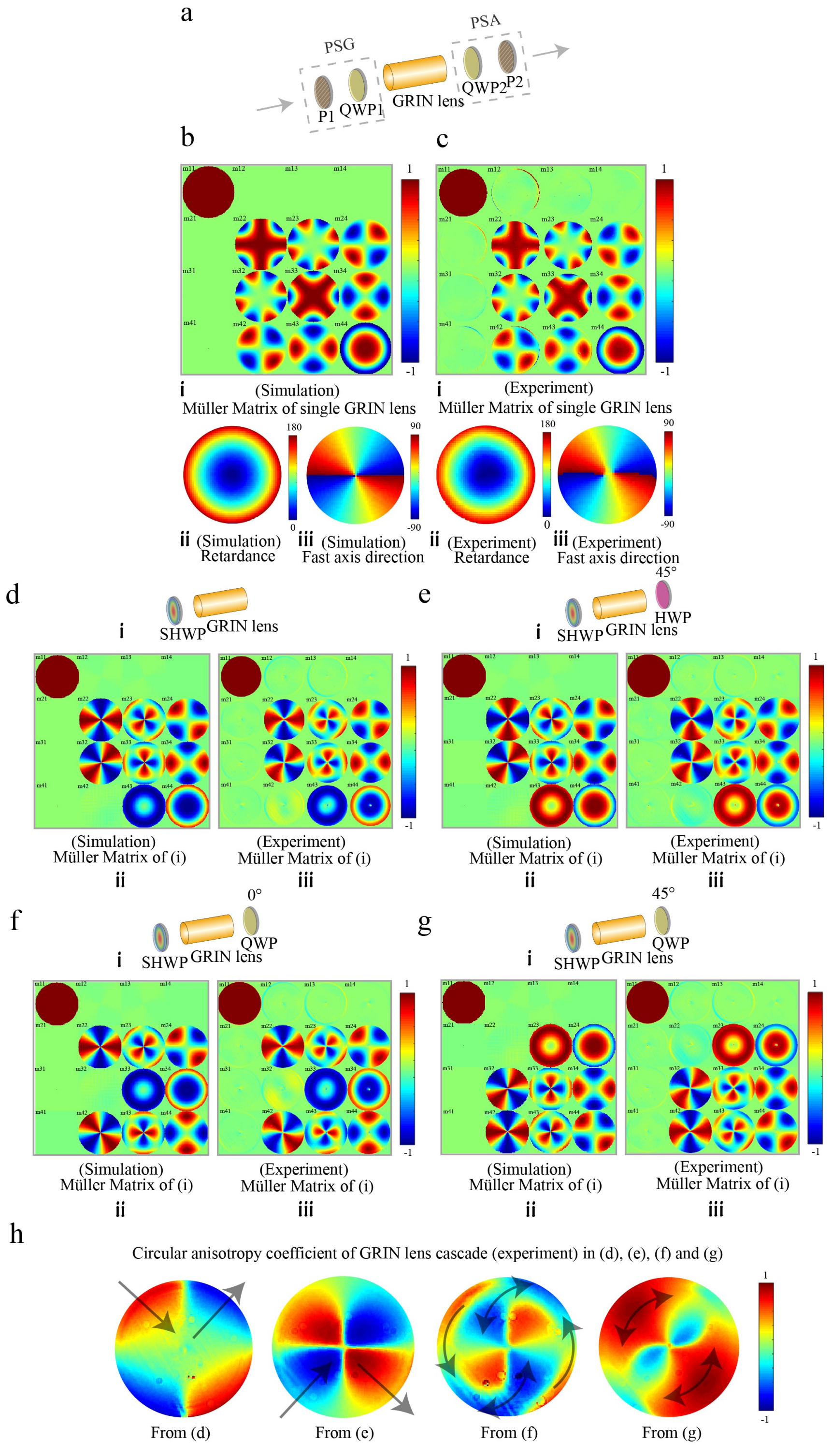

Supplementary Figure 2. Polarization properties of several GRIN lens cascades. (**a**), (**b**) and (**c**) are the schematic of the single GRIN lens cascade and its MM, polarization parameters decomposed by MMPD method both in simulation and experiment. (**d**), (**e**), (**f**), (**g**) are four different SHWP based GRIN lens cascades (the diagrams omit the polarization state generator (PSG) and a polarization state analyser (PSA) parts) with their simulated and experimental MMs. (**h**) Circular anisotropy coefficient of GRIN lens cascade in (**d**), (**e**), (**f**) and (**g**). The grey arrows indicate the anisotropy gradient.

Supplementary Note 2: Modelling of light modulation by the graded birefringence of the GRIN lens

In this section we show mathematically how the retardance profile of the GRIN lens can be calculated and show how the propagation of vector fields through these lenses can be modelled (the equations in this section form the basis for all modelling of the GRIN cascades).

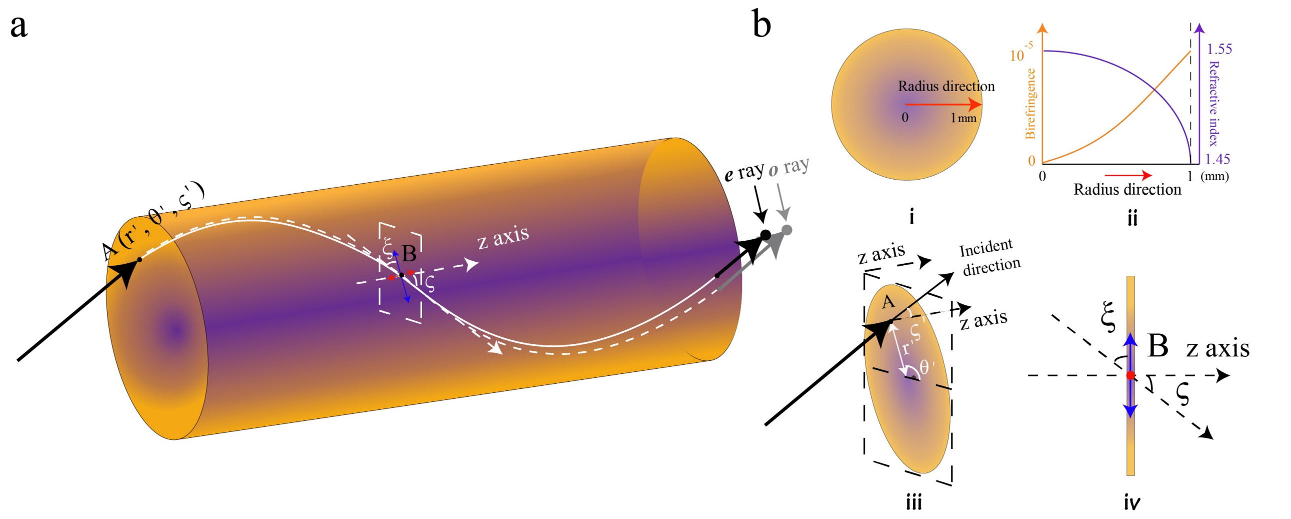

Supplementary Figure 3. Model for analysing the retardance profile of a GRIN lens. (**a**) A specific incident point A is represented by $(r', \theta', \varsigma')$, and the arbitrary point inside GRIN lens along the propagation ray trace is denoted by B (r, θ, ς). The blue double-sided arrow is slow axis direction (extraordinary axis), and the orthometric red counterpart is the fast axis direction (ordinary axis) of that arbitrary point. The black and grey focus points at distal side of the GRIN lens represent focus splitting between the *e* ray (white solid line) and the *o* ray (white dotted line), which is introduced by both the birefringence property and the sinusoid ray trace. (**b**) (i) the section of a GRIN lens shows the refractive index and the birefringence profiles, (ii) shows two curves of these two parameters along the radius direction from centre point to the boundary of GRIN lens, (iii) shows an incident light on point A$(r', \theta', \varsigma')$, on the front surface of GRIN lens, referenced by z axis. (iv) shows the lateral profile of one arbitrary section of GRIN lens, which includes a ray trace passing through point B. ξ is the interior angle between the ray and the extraordinary axis (blue double-sided arrow in (**a**)) at point B, which is the complementary angle to ς, which is the interior angle between the ray and the direction of the z axis.

Supplementary Figure 3a illustrates the refractive index (violet coloured gradient), birefringence (yellow coloured gradient) as well as one arbitrary ray trace (white solid line) when light propagates through the GRIN lens. Supplementary Figure 3b (i) shows the refractive index profile and birefringence profile of a GRIN lens section, and (ii) shows sketches of these two

parameters along the radius direction from the centre to the boundary (refer to the lens from Femto Technology Co. Ltd., G-B161157-S1484). All the calculations below are under the meridional plane approximation[16].

For a general ray within the GRIN lens, the distance from the GRIN lens axis is denoted by r, the azimuth of the incident position is denoted by θ, and the interior angle between the ray and the direction of the z axis is denoted by ς. Then a specific incident ray at point A can be expressed in terms of $(r', \theta', \varsigma')$, also shown in Supplementary Figure 3b (iii). With traditional GRIN lens ray tracing[16], the ray path C (which represents the radius at distance z) of the specific incident beam can be represented by:

$$C(z) = r' \cos(\sqrt{A}z) + \frac{\tan\varsigma'}{\sqrt{A}} \sin(\sqrt{A}z) = \sqrt{r'^2 + \frac{\tan^2\varsigma'}{A}} \cos\left(\sqrt{A}z - \tan^{-1}\left[\frac{\tan\varsigma}{\sqrt{A}r'}\right]\right), \qquad (9)$$

where z is the propagation length along the axis and A is a constant determined by the manufacturing process that is directly related to the refractive index distribution, $\frac{2\pi}{\sqrt{A}}$ is the period of the sinusoidal ray trace and the amplitude is $\sqrt{r'^2 + \frac{\tan^2\varsigma'}{A}}$. The refractive indices seen by the o rays and e rays are denoted by n_o and n_e respectively, which are functions of the radius r. We then could express the refractive index of a GRIN lens in series form to approximate the real distributions:

$$n_o(r) = n_o(0) + \alpha_1 r + \alpha_2 r^2 + \alpha_3 r^3 + \cdots + \alpha_k r^k, \quad (k = 1,2,3,\cdots)$$
$$n_e(r) = n_e(0) + \beta_1 r + \beta_2 r^2 + \beta_3 r^3 + \cdots + \beta_k r^k, \quad (k = 1,2,3,\cdots) \qquad (10)$$

Where $n_o(0)$ and $n_e(0)$ are the refractive indices of the o rays and e rays at the centre (note that $n_o(0) = n_e(0)$), and $\alpha_1, \alpha_2, \alpha_3, \cdots \alpha_k$ and $\beta_1, \beta_2, \beta_3 \cdots \beta_k$ are constants determined by the manufacturing process. The effective refractive index $n_{e'}(r, \xi)$ experienced by the e ray at the local coordinates (r, θ, ς) inside the GRIN lens is represented by:

$$n_{e'}(r, \xi) = \frac{n_e(r) n_o(r)}{\sqrt{n_e^2(r)\cos^2\xi + n_o^2(r)\sin^2\xi}}, \qquad (11)$$

where ξ is the interior angle between the wave normal and the extraordinary axis (blue double-sided arrow in Supplementary Figure 3b (iv)), which is the complementary angle to ς. Along the sinusoidal ray trace, there will be an accumulated phase difference σ between the o rays and e rays when the beam reaches the back surface of the GRIN lens. So, there would be a different overall σ dependent on the traced ray C.

It should be noted here that there would also exist a minor beam split between o rays and e rays due to the birefringence properties of the GRIN lens, with which we are able to modulate the axial resolution of the GRIN lens imaging system as validated in Fig. 3b and 3c in the main article (the point spread function measurement process is adopted with reference to Ref [15]). This splitting is due to the differing sinusoidal optical path lengths of the rays (C_e and C_o respectively). The parameter A in Eq. (9), which is related to the amplitude (and period) of the sinusoidal ray trace, is also different for o rays and e rays (in practice, the parameter should be represented by A_o and A_e, separately, which are determined by the corresponding refractive index profile, Eq. (10)). As the collection of o rays and e rays inside GRIN lens are associated with radial and azimuthal linear polarisation eigenmodes, the corresponding linear polarized light fields play a special role in all GRIN lens based systems. Based upon this understanding, we aim to calculate the optical path length difference (*OPLD*) and the phase difference determined by the retardance σ:

$$OPLD(r,\theta,\varsigma) = \int_{C_e}[n_{e'}(s)]ds - \int_{C_o}[n_o(s)]ds \approx \int_C[n_{e'}(s) - n_o(s)]ds. \tag{12}$$

$$\sigma(r,\theta,\varsigma) = \frac{2\pi}{\lambda}\cdot\int_C[n_{e'}(s) - n_o(s)]ds. \tag{13}$$

Here $n_{e'}(s)$ and $n_o(s)$ are the local refractive indices experienced by e rays and o rays, as a function of distance s along the sinusoidal optical path, as an arc length integral from the original point on the front surface to the exit point on the back surface. Since the birefringence in the GRIN lens is very small, when calculating the accumulated retardance, we can make the approximation that the path of the e rays and the o rays is the same (as shown in Eq. (12)). We use parallel incident light ($\varsigma = 0$ at z = 0) throughout this work, and as the refractive index and birefringence profiles are rotationally symmetric, if we define the wavelength of the incident beam as λ, Eq. (13) would allow us to calculate the corresponding σ.

There are several points that should be considered when estimating the uncertainties of the beam split for different GRIN systems. In this validation work, based upon our polarization measurements above, we estimated that the GRIN lenses we used had maximum birefringence level around 10^{-5}, which is in the range of plausible values for the lithium ion-exchange process used in their manufacture. We tested three different types of pitch 2 GRIN lenses, which, due to their manufacturing processes, had different birefringence properties and lengths. We found that they exhibited variations in beam splitting ranging between ~1.5μm and ~3.0μm. These variations are due to the different optical properties of each lens. We have assumed here

that $n_{\mathrm{o}}(r)$ and $n_{\mathrm{e}}(r)$ follow a parabolic approximation, which one could likely refine for more accurate modelling of the phenomena.

Supplementary Note 3: Experimental set-up for polarization field measurements

Supplementary Figure 4 demonstrates the set-up for polarization field measurements. Here we used a LED light source (633nm, 3mW, $\Delta\lambda$=20nm), a polarizer (P1) and a quarter waveplate (QWP1) to generate a light field of uniform polarization that was incident on the GRIN lens cascade. We used a quarter waveplate (QWP2) and a polarizer (P2) to measure the Stokes vectors of the light field by rotating QWP2 to four different angles[17-19], following the process according to Ref [17-19, 36]. The principal equations for calculation of the Stokes vector light field are shown in Eq. (14) and Eq. (15).

$$S_{\mathrm{out}}^{n} = M_{\mathrm{P2}} M_{\mathrm{QWP2}}^{n} S_{\mathrm{in}}, \ (n = 1,2,3,4 \ldots) \tag{14}$$

$$I = A \cdot S_{\mathrm{in}}, \qquad S_{\mathrm{in}} = A^{-1} \cdot I. \tag{15}$$

where S_{in} is the Stokes vector of the incident light field, M_{P2} and M_{QWP2}^{n} are MMs of the corresponding polarizer and waveplate. S_{out}^{n} is the corresponding output Stokes vector for the nth fast axis orientation state of the quarter waveplate. M_{QWP2}^{n} is the MM of the quarter waveplate for the nth fast axis orientation. A is a $n \times 4$ matrix known as the instrument matrix[19], which is derived from $M_{\mathrm{P2}} \cdot M_{\mathrm{QWP2}}^{n}$. I is the intensity information recorded by the camera.

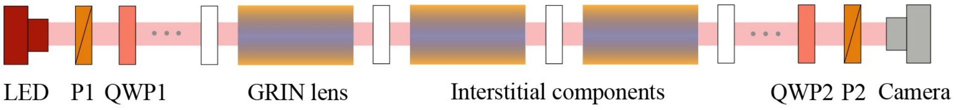

Supplementary Figure 4. Set-up for the polarization field measurement. LED: source; P1, P2: polarizer; QWP1, QWP2: quarter waveplate; Camera: detector. The rest of the structure is the GRIN lens cascade, which combined different kinds of GRIN lenses with interstitial optical elements. Note that imaging optics have been omitted for clarity.

Supplementary Note 4: Theoretical and experimental validation for polarization light field characterization

Vector beams (VB) have attracted great interest for a range of applications that take advantage of their structured polarization[20-22]. They can be used to control the properties of a beam focus in microscopy[23], for the demonstration of Möbius band-like topologies[24], for electron acceleration[25] and material processing[26]. Of particular interest are singular VBs where the polarization distribution in the beam cross section has a vectorial singularity, such as a C-point (where the orientation of the polarization ellipse is undefined) or an L-line (where its handedness is indeterminate)[27, 28]. There is one class of singular VBs called the full Poincaré beam, in which the polarization structure spans the full Poincaré sphere, which are of great interest in many areas of research[27-29]. Here we first characterize the VB components of different vector vortex beams generated by GRIN lens cascade. Following the characterization of the GRIN lens polarization properties[30] using the MM in Supplementary Note 1, the properties can equivalently be represented by the Jones matrix as:

$$J_{\mathrm{GRIN}} = \begin{bmatrix} \sin^2(\theta) + \epsilon\cos^2(\theta) & (\epsilon-1)\sin(\theta)\cos(\theta) \\ (\epsilon-1)\sin(\theta)\cos(\theta) & \cos^2(\theta)+\epsilon\sin^2(\theta) \end{bmatrix}, \tag{16}$$

where $\epsilon = \mathrm{e}^{\mathrm{i}\sigma} = \cos(\sigma) + \mathrm{i}\sin(\sigma)$, and where the desired retardance profile $\sigma = f(r) \propto n_{\mathrm{e}}(r)$ (refer to Supplementary Note 2 for information about $f(r)$ and $n_{\mathrm{e}}(r)$ of the GRIN lens). Here, θ is the slow axis direction of the equivalent waveplate, which is equal to the angle in the cylindrical-coordinate system, r is the radius, σ is the effective cumulative retardance at position $(r,\ \theta)$. If a state of polarization (SOP) incident on the GRIN lens is described by Jones vector $E_{\mathrm{in}} = [\cos\phi, \mathrm{e}^{\mathrm{i}\delta}\sin\phi]^{\mathrm{T}}$, where the parameters ϕ and δ determine the polarization state, then the output polarization beam profile E_{out} is given by $E_{\mathrm{out}} = J_{\mathrm{GRIN}} \cdot E_{\mathrm{in}}$. This can be also represented by Eq. (17):

$$\begin{bmatrix} f_1(\phi,\delta,\theta,\sigma) \\ f_2(\phi,\delta,\theta,\sigma) \end{bmatrix} = \begin{bmatrix} \sin^2(\theta) + \epsilon\cos^2(\theta) & (\epsilon-1)\sin(\theta)\cos(\theta) \\ (\epsilon-1)\sin(\theta)\cos(\theta) & \cos^2(\theta)+\epsilon\sin^2(\theta) \end{bmatrix} \cdot \begin{bmatrix} \cos\phi \\ \mathrm{e}^{\mathrm{i}\delta}\sin\phi \end{bmatrix}, \tag{17}$$

where functions $f_1(\phi,\delta,\theta,\sigma)$ and $f_2(\phi,\delta,\theta,\sigma)$ determine the output VB. As for the case in Fig 2a in the main article, when we apply Eq. (17) to a right hand circular polarized incident beam, $E_{\mathrm{R}} = \frac{1}{\sqrt{2}}[1,\mathrm{i}]^{\mathrm{T}}$, it can be easily validated that when $\sigma = \pi$, the polarization states of the generated beam vary from right-hand circular (at the centre) to left-hand circular (at the outermost ring). Thus, the polarization state of the field $[\,f_1(\phi,\delta,\theta,\sigma)\,, f_2(\phi,\delta,\theta,\sigma)\,]^{\mathrm{T}}$ gradually varies across the transverse plane

from the centre to the outermost boundary such that the field contains all polarization states. This validates that the single GRIN lens cascade can produce a full Poincaré beam for any pure input SOP. Eq. (17) can be similarly used to simulate the effects of other input polarization states. We demonstrated this ability more by using the same single GRIN lens cascade (see Fig. 2a in the main article as well as Supplementary Figure 5a (i) below). Supplementary Figure 5a (ii) shows polarization singularities in the exemplar full Poincaré beam (with topological index[31] η=1), that features C-point and an L-line. The corresponding mapping is shown in Supplementary Figure 5a (iii). A quantitative comparison example for demonstration is presented in Supplementary Figure 5a (iv). The simulation and experimental data are from the beam in Supplementary Figure 5a (ii). A sample distribution of polarizations along the L-line[27, 28] is chosen in order to illustrate the quality of fit; the demonstration on the Poincaré sphere is also given. The mean measurement errors of parameters *S'1, S'2* and *S'3* were 1.8%, 2.1% and 2.7% respectively (normalized by *S'0*). It should be pointed out that the precision benefits from the highly-symmetric birefringence, which is a consequence of the highly symmetric refractive index generated in the manufacturing process. That deserves further exploration, especially if seeking to use GRIN optics as a beam generator for applications that require highly symmetric beams. More examples of beam generation under this cascade are shown in Supplementary Figure 5b through simulation and experiment, which show close correspondence.

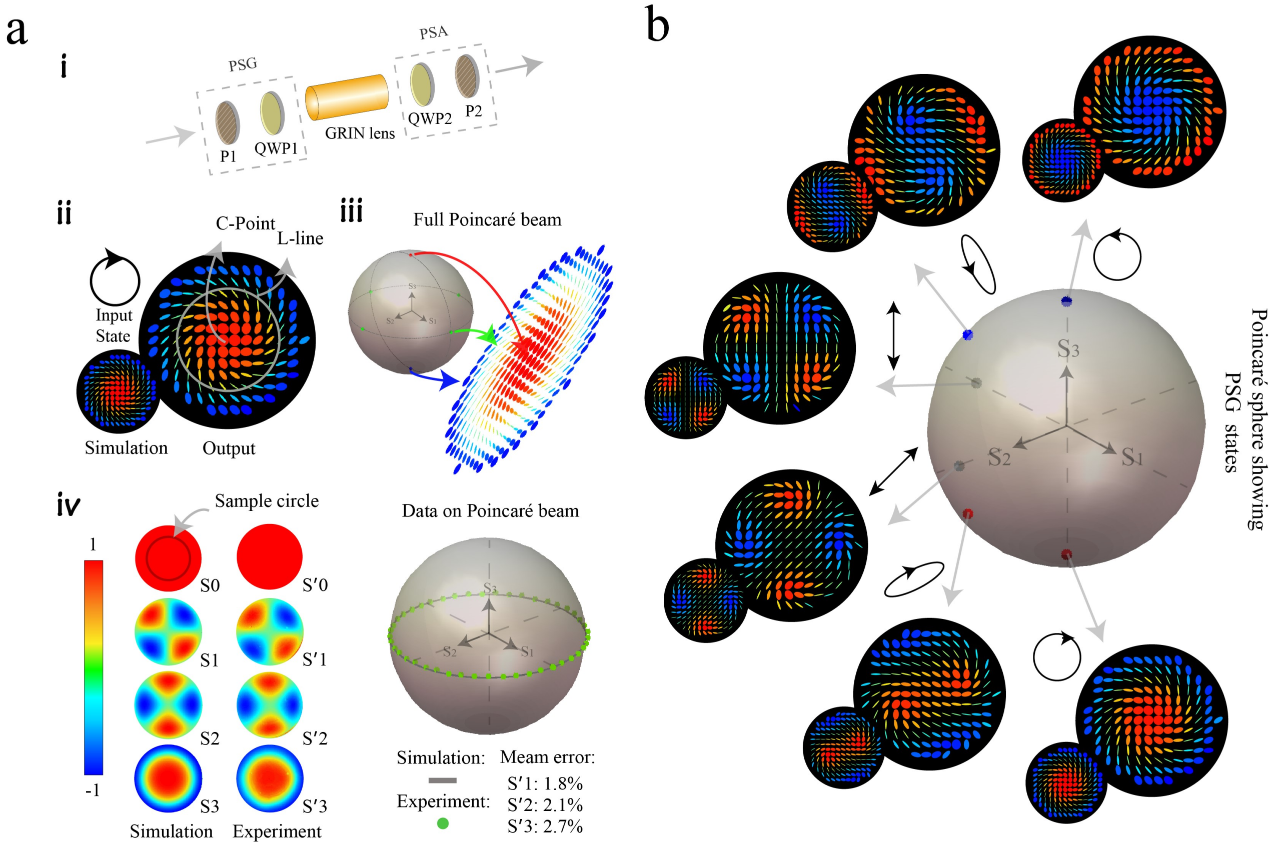

Supplementary Figure 5. Polarization light field generation and characterization. (**a**) (i) Schematic of a single GRIN lens cascade. P1 and P2: fixed polarizers with transmission axis at 0°. QWP1 and QWP2: rotated quarter wave plate. Before the GRIN lens is a PSG that includes P1 and QWP1 to generate a chosen arbitrary polarization state. Following the GRIN lens is a PSA, including P2 and QWP2 that enables Stokes vector measurement. (ii) shows a full Poincaré beam generated by right hand circular input, and its C-point and L-line. The projection of this beam onto Poincaré sphere are demonstrated in (iii). (iv) Stokes vector distribution of the vector beam in (ii) with both simulated and experimental data, alongside the data distribution on the Poincaré sphere. A sample distribution of polarizations along the L-line is chosen for quantitative comparison. The mean measurement errors of parameters *S'1*, *S'2* and *S'3* are given (normalized by *S'0*). (**b**) Beam generated by a single GRIN lens cascade. Five input SOPs generated by the PSG are illustrated (including linear, circular and elliptical states) and shown on the Poincaré sphere. Patterns of polarized light fields in the larger circles represent experimental results and the smaller circles are simulation counterparts. The red side of the color scale indicates right hand circular, whereas blue corresponds to left hand circular.

We further demonstrated and analysed some representative higher-order VBs generated by different cascades – with different combinations of waveplates or different GRIN lenses. An infinite range of cascades can be created, not only through different sequences of GRIN lenses and waveplates, but also through variation of the retardance and fast axis directions of the waveplates. The main principle of VB generation can be found in Eq. (18), where E_{in} is the incident polarization light field and E_{out} is the generated beam profile, both represented by Jones vectors. J_{inter}^{m} and J_{GRIN}^{m} $(m = 1,2,3 \dots)$ are m^{th} Jones

matrices of the interstitial optical elements and the GRIN lenses along the cascade. The simulation approach in this section is established through the methods in Supplementary Note 2 with the combination of Eq. (17) and (18).

$$E_{\text{out}} = J_{\text{inter}}^{m} \cdot J_{\text{GRIN}}^{m} \cdot J_{\text{inter}}^{m-1} \cdots J_{\text{inter}}^{2} \cdot J_{\text{GRIN}}^{1} \cdot J_{\text{inter}}^{1} \cdot E_{\text{in}}. \qquad (m = 1,2,3 \dots) \tag{18}$$

Supplementary Figure 6 shows how different GRIN cascades can be used to generate a range of full Poincaré beams. Supplementary Figure 6a illustrates a type of GRIN lens (coloured red) that has a larger magnitude retardance profile and is almost equivalent to a sequence of three of the first type GRIN lens (shown in yellow, as also used in main article Fig. 2). Supplementary Figure 6b shows the generated beams using right hand circular polarization incident light.

Based on these results in Supplementary Figure 6b, we can make several observations:

1) The GRIN lens cascades are able to generate high radial order VBs due to the nonlinear variation of retardance along the radial direction (Supplementary Figure 6b (ii)), and to create high azimuthal order beams (Supplementary Figure 6b (i)).
2) These beams in Supplementary Figure 6b contains different kinds of topological structures[32-34], including the so-called lemon and star (associated with topological indices $\pm\frac{1}{2}$) as well as spiral (associated with topological index 1), and so on.
3) Supplementary Figures 6b (i) to (iii) show GRIN lens cascade generated multiple full Poincaré beams. In Supplementary Figure 6b (i), there are four sectors in the beam profile, each of which contains a full Poincaré beam (the white and green dotted regions show the boundaries of some of these sectors). In Supplementary Figure 6b (ii), the red dotted circle contains a full Poincaré beam, the beam inside blue dotted circle contains all polarization states spanned on the Poincaré sphere twice. In Supplementary Figure 6b (iii), we find that in a single-beam there are pair of full Poincaré beams, each with an opposite topological charge (the white and green dotted lines show one pair) and opposite handedness on the corresponding topological charge units.
4) The complex full Poincaré beam in Supplementary Figure 6b (iv) contains multiple examples of the same/opposite topological charge units[32-34] and multiple same/opposite handedness beam units (white ellipses demarcate beams that have the same handedness but with opposite topologic charges in solid and dotted outlines; those with green outlines have opposite handedness to those in white outlines but with opposite topologic charge pairs in their own solid and dotted outlines).

To our knowledge, it has not yet been reported that GRIN lenses can act as a beam generator for various types of VBs and especially for full Poincaré beams. All of the above characteristics indicate that the various special beams generated by GRIN lens cascades may open new windows for complex polarization coding and beam engineering.

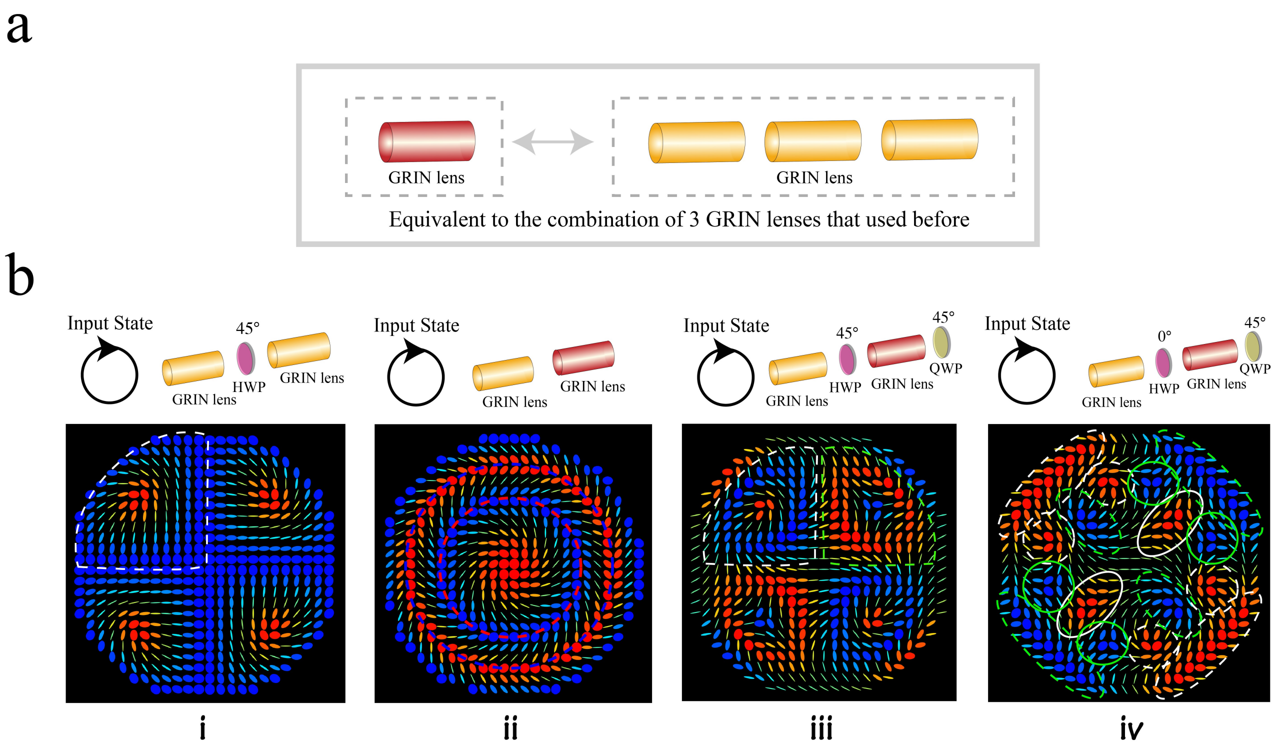

Supplementary Figure 6. Polarization patterns generated by GRIN cascades employing two identical/different GRIN lenses. (**a**) A special GRIN lens used in the later cases that is equivalent to an assembly of three of the GRIN lenses used in the experiment for (**b**). (**b**) are composed of four different cascade structures (from (i) to (iv)) which use identical lenses (yellow one) or different lenses (red one). These cascades were also used in Fig. 2 in the main article. These beams were generated using right hand circular polarized incident light.

Supplementary Note 5: Set-up for interferometry

We employed commonly used interferometric methods[35, 36] to characterize OAM generation. Supplementary Figure 7 illustrates the Mach-Zehnder interferometer, in which we used a He-Ne laser (633nm, 2mW, with a Gaussian intensity distribution), a polarizer (P1) and a quarter waveplate (QWP1) to generate a uniform polarization incident light field at the input to the GRIN lens cascade. The light reflected by the first beam splitter (BS1) into the reference arm, passed off a silver mirror (M1) then was modulated by a half waveplate (HWP1). The beam was expanded then reflected again by another silver mirror (M2) before being combined with the beam from the experimental arm by the second beam splitter (BS2). Finally, the second quarter waveplate (QWP2) and the polarizer (P2) filtered the polarization state of the beam before measurement of the interferogram at the camera.

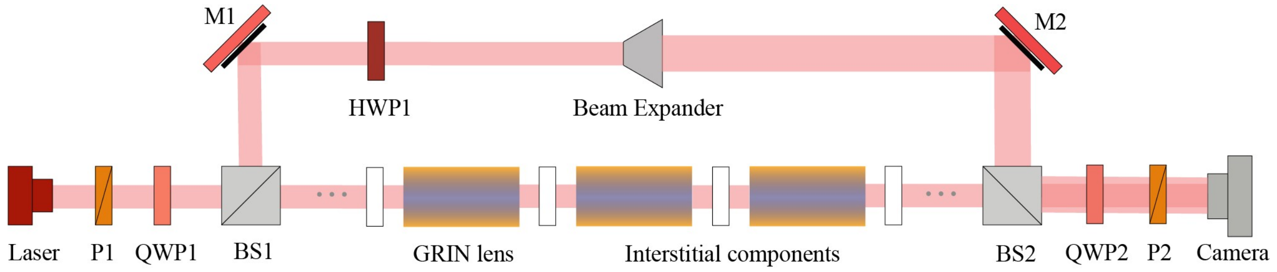

Supplementary Figure 7. Set-up for interferometry. P1, P2: polarizer; QWP1, QWP2: quarter waveplate; HWP1: half waveplate; BS1, BS2: beam splitter; M1, M2: silver mirror; Camera: detector. The rest of the structures comprise the GRIN lens cascade. Note that imaging optics have been omitted for clarity.

Supplementary Note 6: Theoretical and experimental basis of OAM component analysis

A light beam possessing a helicoidal phase-front, described by the structure of $e^{il\vartheta}$, carries OAM of $l\hbar$ (where l is an integer and ħ is the reduced Planck's constant)[37-40]. Since this revelation in 1992[37], various further applications of these beams have been developed, such as optical manipulation[41-44], optical communications[45-47], and in quantum and nano-optics[48-51]. In this section, based on the polarization pattern analysis in above section, we provide further details of the analysis of OAM components of different vector vortex beams generated by GRIN lens cascade. From Eq. (16) we know that the Jones Matrix of a GRIN lens is equal to:

$$J_{\mathrm{GRIN}} = \frac{1}{2}\begin{bmatrix} J_{11} & J_{12} \\ J_{21} & J_{22} \end{bmatrix} = \begin{bmatrix} \sin^2(\theta) + \epsilon\cos^2(\theta) & (\epsilon - 1)\sin(\theta)\cos(\theta) \\ (\epsilon - 1)\sin(\theta)\cos(\theta) & \cos^2(\theta) + \epsilon\sin^2(\theta) \end{bmatrix}, \tag{19}$$

where $\epsilon = \mathrm{e}^{\mathrm{i}\sigma} = \cos(\sigma) + \mathrm{i}\sin(\sigma)$, and $\sigma = f(r) \propto n_{\mathrm{e}}(r)$ (see Supplementary Note 2), θ is the fast axis direction of the equivalent waveplate which equals to the azimuthal angle in the cylindrical coordinate system, r is the radius across the section of the GRIN lens, σ is the linear retardance value of the equivalent waveplate at the position (r, θ). For simplicity of notation, let us make $S^{2\theta} = \sin(2\theta)$, $C^{2\theta} = \cos(2\theta)$, $S^{\sigma} = \sin(\sigma)$, $C^{\sigma} = \cos(\sigma)$. From this we obtain:

$$\begin{bmatrix} J_{11} & J_{12} \\ J_{21} & J_{22} \end{bmatrix} = \begin{bmatrix} 1 + C^{\sigma} + C^{2\theta}(C^{\sigma} - 1) + S^{\sigma}(1 + C^{2\theta})\mathrm{i} & S^{2\theta}(C^{\sigma} - 1) + S^{\sigma}S^{2\theta}\mathrm{i} \\ S^{2\theta}(C^{\sigma} - 1) + S^{\sigma}S^{2\theta}\mathrm{i} & 1 + C^{\sigma} + C^{2\theta}(1 - C^{\sigma}) + S^{\sigma}(1 - C^{2\theta})\mathrm{i} \end{bmatrix}. \tag{20}$$

Suppose a uniformly polarized beam represented by Jones vector E_{in} passes through the GRIN lens J_{GRIN}. We now examine the properties of the generated vector $E_{\mathrm{out}} = J_{\mathrm{GRIN}} \cdot E_{\mathrm{in}}$ in left- and right-circular eigenpolarization bases: $E_{\mathrm{L}} = \frac{1}{\sqrt{2}}[1, -\mathrm{i}]^{\mathrm{T}}$, $E_{\mathrm{R}} = \frac{1}{\sqrt{2}}[1, \mathrm{i}]^{\mathrm{T}}$. Following the OAM generation case in the main article, we used a fixed input SOP: $E_{\mathrm{R}} = \frac{1}{\sqrt{2}}[1, \mathrm{i}]^{\mathrm{T}}$ entering a single GRIN lens. Using the Jones matrix calculation of Eq. (20), we find that,

$$J_{\mathrm{GRIN}}\begin{bmatrix} 1 \\ \mathrm{i} \end{bmatrix} = z_1\begin{bmatrix} 1 \\ \mathrm{i} \end{bmatrix} + z_2\begin{bmatrix} 1 \\ -\mathrm{i} \end{bmatrix}, \tag{21}$$

where z_1 and z_2 can be written as $z_1 = \frac{1}{2}(\mathrm{e}^{\mathrm{i}0} + \mathrm{e}^{\mathrm{i}\sigma})$, $z_2 = \frac{1}{2}\left(\mathrm{e}^{\mathrm{i}(2\theta+\pi)} + \mathrm{e}^{\mathrm{i}(2\theta+\sigma)}\right)$. So, if we use a PSA for state $E_{\mathrm{L}} = \frac{1}{\sqrt{2}}[1, -\mathrm{i}]^{\mathrm{T}}$, would only see the $z_2 E_{\mathrm{L}}$ term from Eq. (21), which can be written as,

$$z_2 \begin{bmatrix} 1 \\ -\mathrm{i} \end{bmatrix} = \frac{1}{2}\left(\mathrm{e}^{\mathrm{i}(2\theta+\pi)} + \mathrm{e}^{\mathrm{i}(2\theta+\sigma)}\right) \begin{bmatrix} 1 \\ -\mathrm{i} \end{bmatrix} = A\, \mathrm{e}^{\mathrm{i}(2\theta+\varphi)} \begin{bmatrix} 1 \\ -\mathrm{i} \end{bmatrix}, \tag{22}$$

where $A(r)$ is the amplitude distribution. When considering OAM, we are only concerned about the phase term $(2\theta + \varphi)$ in Eq. (22), where $\varphi(r)$ is an initial phase delay determined by the retardance profile $\sigma(r)$ (see Supplementary Note 2). The exponent $\mathrm{i}(2\theta)$ shows that the analyzed E_{L} beam exhibits two units of OAM, which reveals that the GRIN lens can be used as a spin-to-orbital angular momentum convertor. Corresponding results can be found in Fig. 2a (iii) to (v), Supplementary Figures 8a and 8c.

We now examine the properties of the generated vector $E_{\mathrm{out}} = J_{\mathrm{GRIN}} \cdot E_{\mathrm{in}}$ in another two specific eigenpolarization bases: $E_{\mathrm{H}} = [1{,}0]^{\mathrm{T}}$, $E_{\mathrm{V}} = [0{,}1]^{\mathrm{T}}$. We used a fixed input SOP: $E_{\mathrm{R}} = \frac{1}{\sqrt{2}}[1, \mathrm{i}]^{\mathrm{T}}$ entering a single GRIN lens:

$$J_{\mathrm{GRIN}} \begin{bmatrix} 1 \\ \mathrm{i} \end{bmatrix} = z_{1'} \begin{bmatrix} 1 \\ 0 \end{bmatrix} + z_{2'} \begin{bmatrix} 0 \\ 1 \end{bmatrix}. \tag{23}$$

From calculation, $[z_{1'} \quad z_{2'}]$ can be written as exponential form:

$$z_{1'} = \frac{1}{2}\left(\mathrm{e}^{\mathrm{i}0} + \mathrm{e}^{\mathrm{i}\sigma} + \mathrm{e}^{\mathrm{i}(2\theta+\pi)} + \mathrm{e}^{\mathrm{i}(\sigma+2\theta)}\right), \tag{24}$$

$$z_{2'} = \frac{1}{2}\left(\mathrm{e}^{\mathrm{i}\left(\frac{\pi}{2}\right)} + \mathrm{e}^{\mathrm{i}\left(\sigma+\frac{\pi}{2}\right)} + \mathrm{e}^{\mathrm{i}\left(2\theta+\frac{\pi}{2}\right)} + \mathrm{e}^{\mathrm{i}\left(\sigma+2\theta-\frac{\pi}{2}\right)}\right). \tag{25}$$

In Supplementary Figure 8 below, the corresponding phase profiles, intensity profiles, as well as interference patterns using analyzers described by $[1, 0]^{\mathrm{T}}$ (both in experiment and simulation) can be found in Supplementary Figures 8d (iii) to 8h (iii) and 8d (vii) to 8h (vii). We also modified the PSA to obtain a wider variety of wave fronts for illustration; the sequence is shown in Supplementary Figure 8b on the pathway from (i) to (ix) on the Poincaré sphere of the PSA state. The simulated and experimental interferograms as well as intensity distributions can be found in Supplementary Figures 8d to 8h.

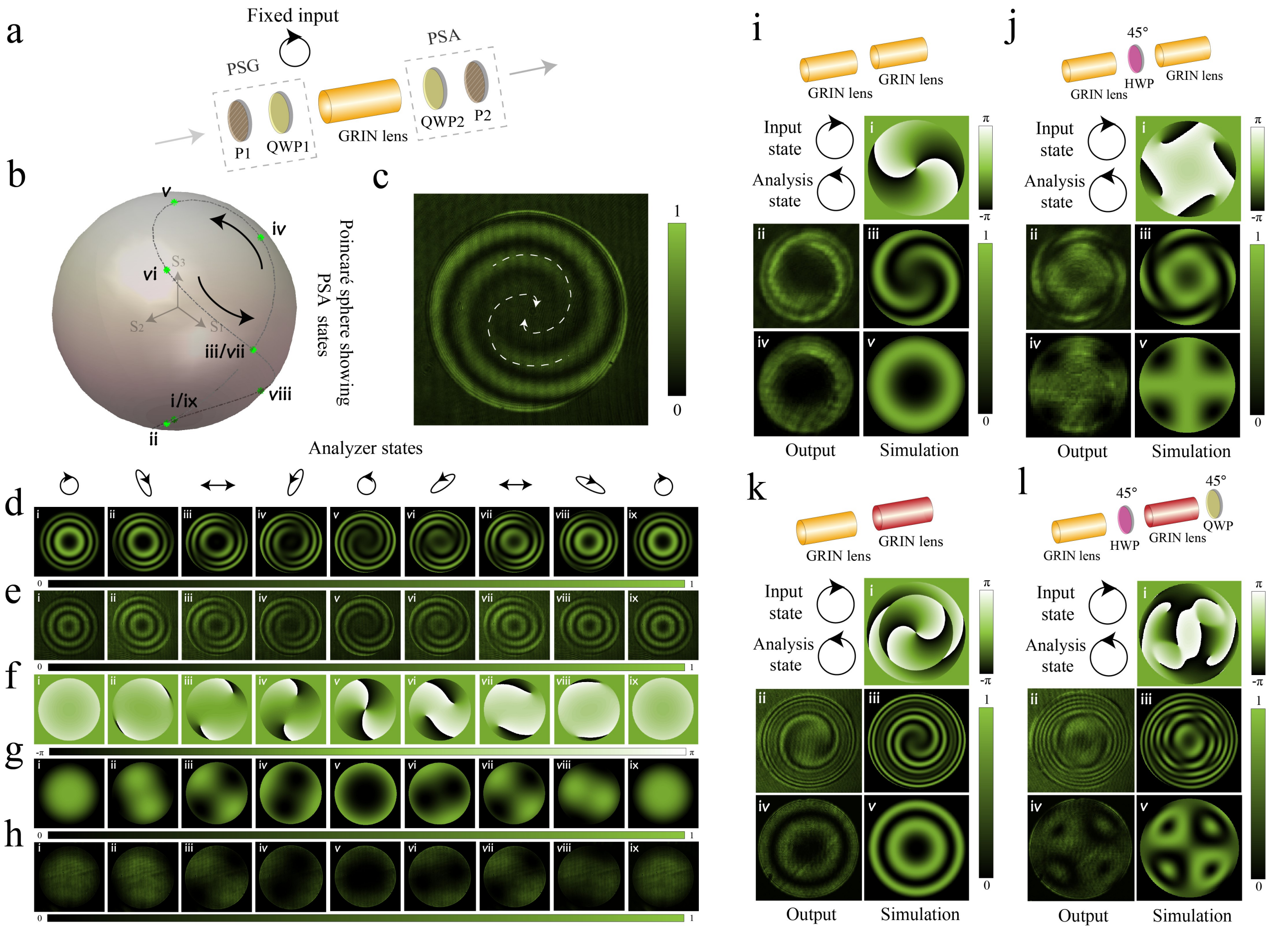

Supplementary Figure 8. OAM generation and characterization. (**a**) Simplified setup for the OAM generation validation. P1 and P2: fixed polarizers with transmission axis at 0°. QWP1: fixed quarter wave plate with fast axis orientation at 45°. QWP2: rotating quarter wave plate. The PSG comprises P1 and QWP1 to generate fix circular SOP, and the PSA is constructed by P2 and QWP2. (**b**) A Poincaré sphere representation of the pathway of detection polarization states (i) to (ix) in (**d**) to (**h**). (**c**) A particular illustration of the interference pattern of state (*v*) in (**f**). The two spirals indicated that the light beam contained two units of OAM. (**d**) to (**h**) Interference patterns, phase profiles, intensity distributions under different fast axis orientations of the second quarter waveplate (QWP2 in (**a**)) from -45° to 135° with interval 22.5°. The rows show: (**d**) Simulation of interference patterns. (**e**) Experimental interference patterns. (**f**) Simulation of phase patterns. (**g**) Simulation of intensity distribution. (**h**) Experimental intensity distribution. The analyser states for each column are shown above (**b**). The data here are normalized besides the simulation phase value. (**i**), (**j**), (**k**) and (**l**) are higher-order GRIN lens cascades and their generated complex phase patterns, interference patterns and intensity profiles under specific input SOP and analysis polarization state. Note that the PSG and PSA parts have been omitted for clarity, and that in (**i**) and (**j**) the second GRIN lens (yellow) is of the same type as the first lens, whereas in (**k**) and (**l**) GRIN lens (red) has a larger magnitude retardance profile, as used in the previous section.

Similar to the polarization fields shown in the main article (Fig. 2), some demonstrations of the complex phase modulation abilities of higher order GRIN lens cascades are also shown in Supplementary Figures 8i to 8l. It can be seen that higher order cascades are able to generate different complex phase patterns that also feature higher order OAM components by choosing the input SOP and the analysis polarization state. An example can been seen in Supplementary Figure 8j, which shows four discrete singularities. This method can benefit various applications requiring phase control, especially for OAM or vortex beam related applications.

We further demonstrated cases using linear polarized incident eigenpolarization bases: $E_{\mathrm{H}} = [1{,}0]^{\mathrm{T}}$, $E_{\mathrm{V}} = [0{,}1]^{\mathrm{T}}$. We first used a fixed input SOP: $E_{\mathrm{H}} = [1{,}0]^{\mathrm{T}}$ entering a single GRIN lens cascade, and analyzed by $E_{\mathrm{H}} = [1{,}0]^{\mathrm{T}}$ and $E_{\mathrm{V}} = [0{,}1]^{\mathrm{T}}$.

$$J_{\mathrm{GRIN}}\begin{bmatrix}1\\0\end{bmatrix} = z_{1''}\begin{bmatrix}1\\0\end{bmatrix} + z_{2''}\begin{bmatrix}0\\1\end{bmatrix}. \tag{26}$$

We calculate $z_{1''}$ and $z_{2''}$ by through the same process used before, as shown in Eq. (27) and (28). In the following Supplementary Figures 9a and 9b we demonstrate phase profiles, intensity distributions and interference patterns (where the object beam is interfered with the same SOP light).

$$z_{1''} = \begin{cases} \frac{1}{2}\left(\mathrm{e}^{\mathrm{i}0} + \mathrm{e}^{\mathrm{i}\sigma}\right) + \frac{1}{2}|\cos(2\theta)|\left(\mathrm{e}^{\mathrm{i}0} + \mathrm{e}^{\mathrm{i}(\sigma+\pi)}\right), & \theta \in \left[-\frac{3\pi}{4}, -\frac{\pi}{4}\right] \cup \left[\frac{\pi}{4}, \frac{3\pi}{4}\right], \\ \frac{1}{2}\left(\mathrm{e}^{\mathrm{i}0} + \mathrm{e}^{\mathrm{i}\sigma}\right) + \frac{1}{2}|\cos(2\theta)|\left(\mathrm{e}^{\mathrm{i}\pi} + \mathrm{e}^{\mathrm{i}\sigma}\right), & \text{Otherwise.} \end{cases} \tag{27}$$

$$z_{2''} = \begin{cases} \frac{1}{2}|\sin(2\theta)|\left(\mathrm{e}^{\mathrm{i}0} + \mathrm{e}^{\mathrm{i}(\sigma+\pi)}\right), & \theta \in \left[-\frac{\pi}{2}, 0\right] \cup \left[\frac{\pi}{2}, \pi\right], \\ \frac{1}{2}|\sin(2\theta)|\left(\mathrm{e}^{\mathrm{i}\pi} + \mathrm{e}^{\mathrm{i}\sigma}\right), & \text{Otherwise.} \end{cases} \tag{28}$$

Using the basis $E_{\mathrm{R}} = \frac{1}{\sqrt{2}}[1, \mathrm{i}]^{\mathrm{T}}$, $E_{\mathrm{L}} = \frac{1}{\sqrt{2}}[1, -\mathrm{i}]^{\mathrm{T}}$ as the analysis eigenbasis, we obtain:

$$J_{\mathrm{GRIN}}\begin{bmatrix}1\\0\end{bmatrix} = z_{1'''}\begin{bmatrix}1\\\mathrm{i}\end{bmatrix} + z_{2'''}\begin{bmatrix}1\\-\mathrm{i}\end{bmatrix}, \tag{29}$$

from which we derive Eq. (30) and Eq. (31) below. The corresponding phase profiles, intensity distributions and interference patterns can be found in Supplementary Figures 9c and 9d.

$$z_{1'''} = \frac{1}{4}\left(\mathrm{e}^{\mathrm{i}0} + \mathrm{e}^{\mathrm{i}\sigma} + \mathrm{e}^{-\mathrm{i}(2\theta+\pi)} + \mathrm{e}^{\mathrm{i}(\sigma-2\theta)}\right), \tag{30}$$

$$z_{2'''} = \frac{1}{4}\left(\mathrm{e}^{\mathrm{i}0} + \mathrm{e}^{\mathrm{i}\sigma} + \mathrm{e}^{\mathrm{i}(2\theta+\pi)} + \mathrm{e}^{\mathrm{i}(\sigma+2\theta)}\right). \tag{31}$$

The simulations in this section were based on the above theoretical equations. It should be noted that other combinations of incident polarizations or analysis eigenvectors, or even higher order GRIN lens cascades cases (combined with Eq. (18)), can be easily calculated through the same process above.

We have demonstrated here that the GRIN lens cascades have the potential to generate complex structured phase profiles under different input/output eigenpolarizations. This novel structure for phase modulation (including the generation of OAM), derives from the special birefringence profile of GRIN optics. To our knowledge, this has not yet been reported. It might pave the way for new methods of complex beam engineering and benefit corresponding applications such as OAM related quantum and nano-optics.

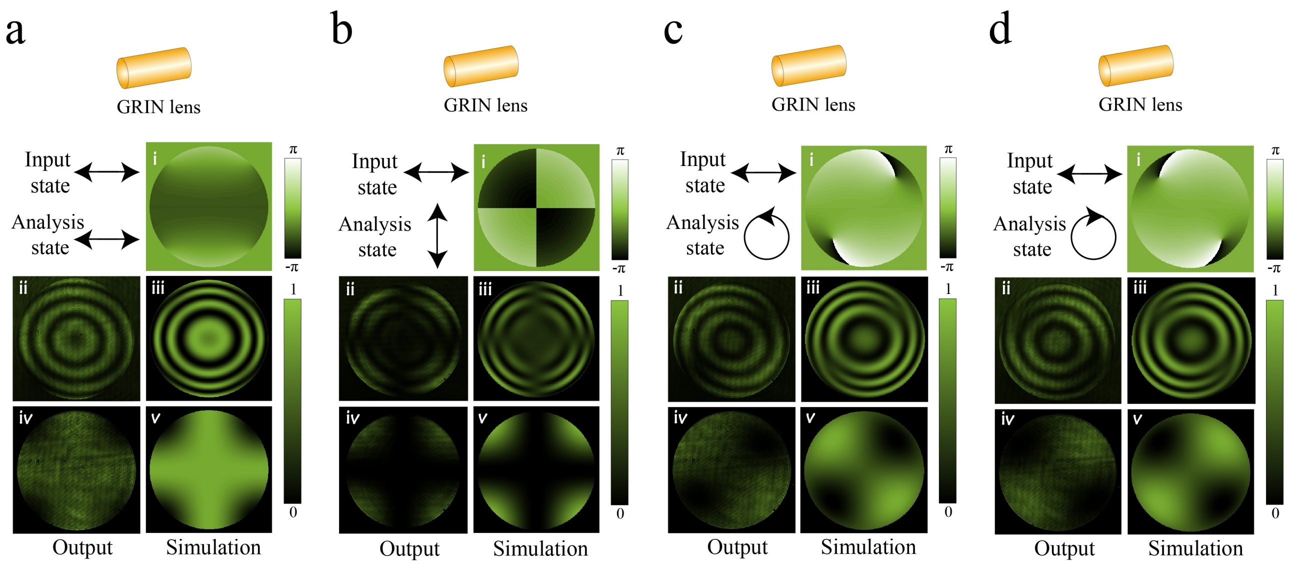

Supplementary Figure 9. Phase profiles, interference patterns, intensity distributions generated by the single GRIN lens. (**a**), (**b**), (**c**) and (**d**) are phase and intensity information extracted using horizontal linear polarized light at the input and analysis through: (**a**) horizontal linear, (**b**) vertical linear, (**c**) left hand circular and (**d**) right hand circular polarized light, respectively. In each group of four images, (i) shows simulated absolute phase profiles, (ii) and (iii) are experimental and simulated interference patterns, (iv) and (v) are experimental and simulated intensity distributions. For clarity, the PSG and PSA parts are not shown in the cascades.

Supplementary Note 7: Optimization of the polarisation state generator (PSG) and polarisation state analyser (PSA)

There are many ways to specify the illumination polarizations and analyser configurations for MM polarimetry[1, 3-5, 7-11, 13, 18, 52, 53]. To find optimum measurement configurations, one can use the condition number (CN) of the system as an optimisation criterion for both the PSG and PSA. The CN can also be related to the volume of the inner tetrahedroid constructed by the SOPs inside the Poincaré sphere (shown in Fig. 4b (ii) of the main article)[18, 52].

Based upon this, we chose a PSG that consisted of four sectors of differently oriented quarter waveplates, as shown in Fig. 4b (i) in the main article, where the fast axes of waveplates 1 to 4 were oriented at 45°, 15.9°, 74.1°, and -45° according to the optimization shown in Ref [52]. Similar CN analysis has shown that the 132° phase retardance with four different fast axis directions is optimal for the PSA[18, 52], providing a CN of 1.732. As shown in Fig. 4c (ii) and 4c (iii) in the main article, the polarization properties of the GRIN lens cascade have rotational symmetry (this cascade is also used to generate multiple full Poincaré beams units in a single beam in Supplementary Note 4). However, each of the four sectors equivalently encompasses required optimal combinations of retardance and fast axis direction, and each sector is enabled to generate a full Poincaré beam. Additionally, as the birefringence profile is determined by the atomic level scale of ion implantation, the number of potential channels in the system is determined in effect by the pixelation of the camera. Thus, the GRIN lens based MM polarimeter has the great advantage of providing multiple parallel channels to aid the calculation. We chose m (m=4, 5, 6…) pixels in each channel to calculate four Stokes vectors simultaneously, to then work out the target MM. If taking MMPD results as a standard, the chosen pixels encompass the analysis channels of the 132° phase retardance ring on each PSA sector, so it follows that the optimum CN (1.732) can be achieved. The additional effective pixels can help reduce spatial noise when combining the pixel information[18]. For these reasons, the GRIN cascade can form as a basis of an optimal MM measurement system.

Supplementary Note 8: Measurement of the Müller matrix (MM) with the single shot polarimeter

We derive here the main equations describing the principle of operation of the MM polarimeter. We use *n* (*n*=1, 2, 3 and 4) to denote the four areas of the PSG array corresponding to the four sectors of the FQWP, and *m* (*m*=1, 2, 3 ... *M*) to denote the chosen pixel number in each sector.

$$S_{\mathrm{out}}^{n,m} = M_{\mathrm{P2}} \cdot M_{\mathrm{GRIN2}}^{n,m} \cdot M_{\mathrm{HWP}} \cdot M_{\mathrm{GRIN1}}^{n,m} \cdot M_{\mathrm{Sample}} \cdot S_{\mathrm{in}}^{n,m}, \tag{32}$$

where $S_{\mathrm{in}}^{n,m}$ represents incident Stokes vectors generated by the four sectors of the PSG array (the *m* is the chosen pixel number in each sector; all pixels in each sector have same incident SOP). $S_{\mathrm{out}}^{n,m}$ is the combination of output Stokes vectors (both in the channel *m* of the sector *n*; the same meanings are followed in the later description). M_{Sample} denotes the MM of the targeted sample, and M_{P2}, M_{HWP} denote the MMs of the polarizer and the half wave plate. $M_{\mathrm{GRIN1}}^{n,m}$, $M_{\mathrm{GRIN2}}^{n,m}$ are MMs of the GRIN lenses in the corresponding spatial positions. Since only intensity information can be recorded by the camera, which means only the first element of $S_{\mathrm{out}}^{n,m}$ can be obtained (the intensity information at sector *n* from channel *m*), then Eq. (32) can be modified into Eq. (33) and Eq. (34):

$$I_{\mathrm{out}} = A \cdot M_{\mathrm{Sample}} \cdot S_{\mathrm{in}}, \tag{33}$$

$$\begin{bmatrix} I_{\mathrm{out}}^{1,1} \\ \vdots \\ I_{\mathrm{out}}^{1,M} \\ I_{\mathrm{out}}^{2,1} \\ \vdots \\ I_{\mathrm{out}}^{2,M} \\ I_{\mathrm{out}}^{3,1} \\ \vdots \\ I_{\mathrm{out}}^{3,M} \\ I_{\mathrm{out}}^{4,1} \\ \vdots \\ I_{\mathrm{out}}^{4,M} \end{bmatrix} = \begin{bmatrix} a_0^{1,1} & a_1^{1,1} & a_2^{1,1} & a_3^{1,1} \\ & & \vdots & \\ a_0^{1,M} & a_1^{1,M} & a_2^{1,M} & a_3^{1,M} \\ a_0^{2,1} & a_1^{2,1} & a_2^{2,1} & a_3^{2,1} \\ & & \vdots & \\ a_0^{2,M} & a_1^{2,M} & a_2^{2,M} & a_3^{2,M} \\ a_0^{3,1} & a_1^{3,1} & a_2^{3,1} & a_3^{3,1} \\ & & \vdots & \\ a_0^{3,M} & a_1^{3,M} & a_2^{3,M} & a_3^{3,M} \\ a_0^{4,1} & a_1^{4,1} & a_2^{4,1} & a_3^{4,1} \\ & & \vdots & \\ a_0^{4,M} & a_1^{4,M} & a_2^{4,M} & a_3^{4,M} \end{bmatrix} \cdot M_{\mathrm{Sample}} \cdot \begin{bmatrix} s_0^{1,1} & \cdots & s_0^{1,M} & s_0^{2,1} & \cdots & s_0^{2,M} & s_0^{3,1} & \cdots & s_0^{3,M} & s_0^{4,1} & \cdots & s_0^{4,M} \\ \vdots & \ddots & \vdots & \vdots & \ddots & \vdots & \vdots & \ddots & \vdots & \vdots & \ddots & \vdots \\ s_3^{1,1} & \cdots & s_3^{1,M} & s_3^{2,1} & \cdots & s_3^{2,M} & s_3^{3,1} & \cdots & s_3^{3,M} & s_3^{4,1} & \cdots & s_3^{4,M} \end{bmatrix}. \tag{34}$$

Where the intensity information recorded by the camera for each modulation and detection channel are defined as a vector:

$$I_{\mathrm{out}} = \begin{bmatrix} I_{\mathrm{out}}^{1,1} & \cdots & I_{\mathrm{out}}^{1,M} & I_{\mathrm{out}}^{2,1} & \cdots & I_{\mathrm{out}}^{2,M} & I_{\mathrm{out}}^{3,1} & \cdots & I_{\mathrm{out}}^{3,M} & I_{\mathrm{out}}^{4,1} & \cdots & I_{\mathrm{out}}^{4,M} \end{bmatrix}^{\mathrm{T}}, \tag{35}$$

and A is defined by:

$$A = [A^{1,1} \quad \cdots \quad A^{1,M} \quad A^{2,1} \quad \cdots \quad A^{2,M} \quad A^{3,1} \quad \cdots \quad A^{3,M} \quad A^{4,1} \quad \cdots \quad A^{4,M}]^{\mathrm{T}}, \tag{36}$$

which is a $4m \times 4$ matrix in which each component $A^{n,m} = [a_0^{n,m} \quad a_1^{n,m} \quad a_2^{n,m} \quad a_3^{n,m}]$ consists of the first row of the corresponding $M_{\mathrm{P2}} \cdot M_{\mathrm{GRIN2}}^{n,m} \cdot M_{\mathrm{HWP}} \cdot M_{\mathrm{GRIN1}}^{n,m}$. We then expand $S_{\mathrm{in}}^{n,m} = [s_0^{n,m} \quad s_1^{n,m} \quad s_2^{n,m} \quad s_3^{n,m}]^{\mathrm{T}}$, and let

$$S_{\mathrm{in}} = \begin{bmatrix} s_0^{1,1} & \cdots & s_0^{1,M} & s_0^{2,1} & \cdots & s_0^{2,M} & s_0^{3,1} & \cdots & s_0^{3,M} & s_0^{4,1} & \cdots & s_0^{4,M} \\ \vdots & \ddots & \vdots & \vdots & \ddots & \vdots & \vdots & \ddots & \vdots & \vdots & \ddots & \vdots \\ s_3^{1,1} & \cdots & s_3^{1,M} & s_3^{2,1} & \cdots & s_3^{2,M} & s_3^{3,1} & \cdots & s_3^{3,M} & s_3^{4,1} & \cdots & s_3^{4,M} \end{bmatrix}, \tag{37}$$

which is a $4 \times 4m$ matrix that consists of columns that are individual Stokes vectors from each combination of PSG sectors and camera pixels. Then the MM of the sample can be calculated as:

$$M_{\mathrm{Sample}} = A^{-1} \cdot I_{\mathrm{out}} \cdot S_{\mathrm{in}}^{-1}, \tag{38}$$

where S_{in}^{-1} is the pseudo inverse matrix of S_{in} , and A^{-1} is the pseudo inverse matrix of A.

Supplementary Note 9: Validation of feasibility using standard samples

In order to test the capabilities of the MM polarimeter, we used as samples four polarizers with different orientations (0°, 90°, 45°, -45°), which are illustrated as P2 to P5 in the experimental setup in Supplementary Figure 10a. We took measurements of each polarizer in a single-shot and compared the derived MM elements with the ground truth MMs (Supplementary Figures 10b (i) to 10b (iv)). The maximum errors of the derived MM elements were smaller than 6.93%. The GRIN lens cascade based polarimeter has the following advantages: 1) it can perform single-shot MM measurement in a robust and stable manner; 2) single-shot measurements enable precise measurement of moving/changing objects; 3) it has the potential to be miniaturised into an integrated instrument, especially a fibre-based probe with scanning detection for clinical applications.

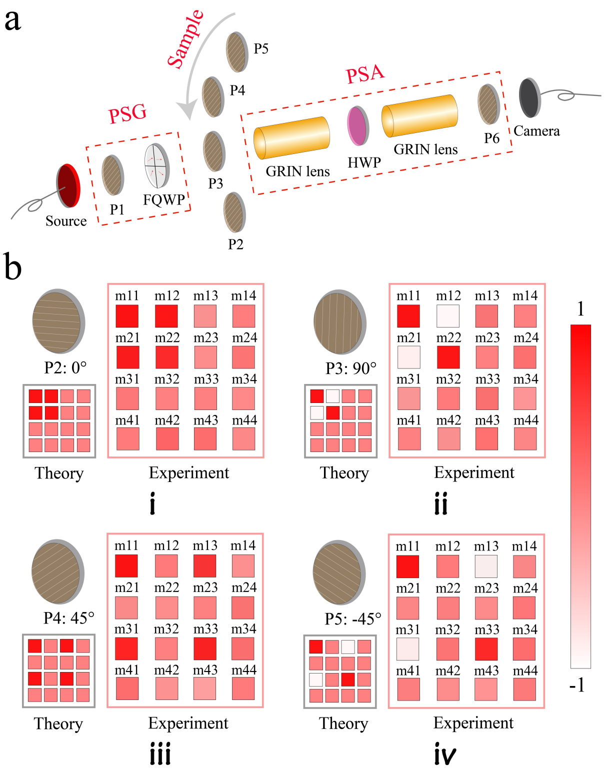

Supplementary Figure 10. Characterization of the GRIN lens based MM polarimeter. (**a**) Simplified setup for the validation experiment of the polarimeter. PSG: P1, polarizer; FQWP, four-quadrant quarter waveplate, with different fast axis orientations in each quadrant. PSA: P6, polarizer; HWP, half waveplate. P2 to P5: polarizers with transmission axis orientations at 0°, 90°, 45°, and -45°. (**b**) (i) to (iv) The measured single-shot MMs of the four polarizers (red boxes), and corresponding ground truth (black boxes).

Supplementary Note 10: Tissue sample information, sampling method and statistical analysis for tissue measurements

Clinically, different stages of breast ductal carcinoma tissues have different proportions and distribution of fibrous structures in and around the milk ducts (there are stages 1 (normal), 2 (ductal carcinoma in situ) and 3 (invasive ductal carcinoma))[53]. Through this work, the samples were unstained, dewaxed sections of human breast ductal carcinoma tissue slices at different stages. They were prepared and provided by the Department of Pathology, Shenzhen Sixth People's (Nanshan) Hospital. For the tissues in stages 1 (normal), and 3 (invasive ductal carcinoma), we selected 12-μm-thick slices[53] for demonstration. For comparison, the corresponding 4-μm-thick hematoxylin and eosin (H and E) stained slices[53] were also prepared. The age range of the patients was from thirty to fifty-five years. This work was approved by the Ethics Committee of the Shenzhen Sixth People's (Nanshan) Hospital.

We measured 10 points per sample using our new MM polarimeter and a conventional MM microscope (overall 20 samples), as ground truth for quantitative comparison. In each sample, we chose the points by using MM polarimeter with the diameter of the field of view (FOV) at 0.19mm (Femto Technology Co. Ltd., G-B151157-S1483). The FOV of the MM microscope (calibration precision < 0.3%) was around four times larger than that of the polarimeter, so we took measurements from chosen sub-areas within same FOV of MM polarimeter to be processed. Then we calculated the mean value of the retardance across these areas to set as ground truth for further comparison through the same quantitative comparison process used to differentiate the breast cancerous stages by polarization parameters[53]. Example MMs, as well as corresponding MMPD parameters, from healthy or cancerous tissue are illustrated alongside quantitative statistic histograms in Supplementary Figure 11.

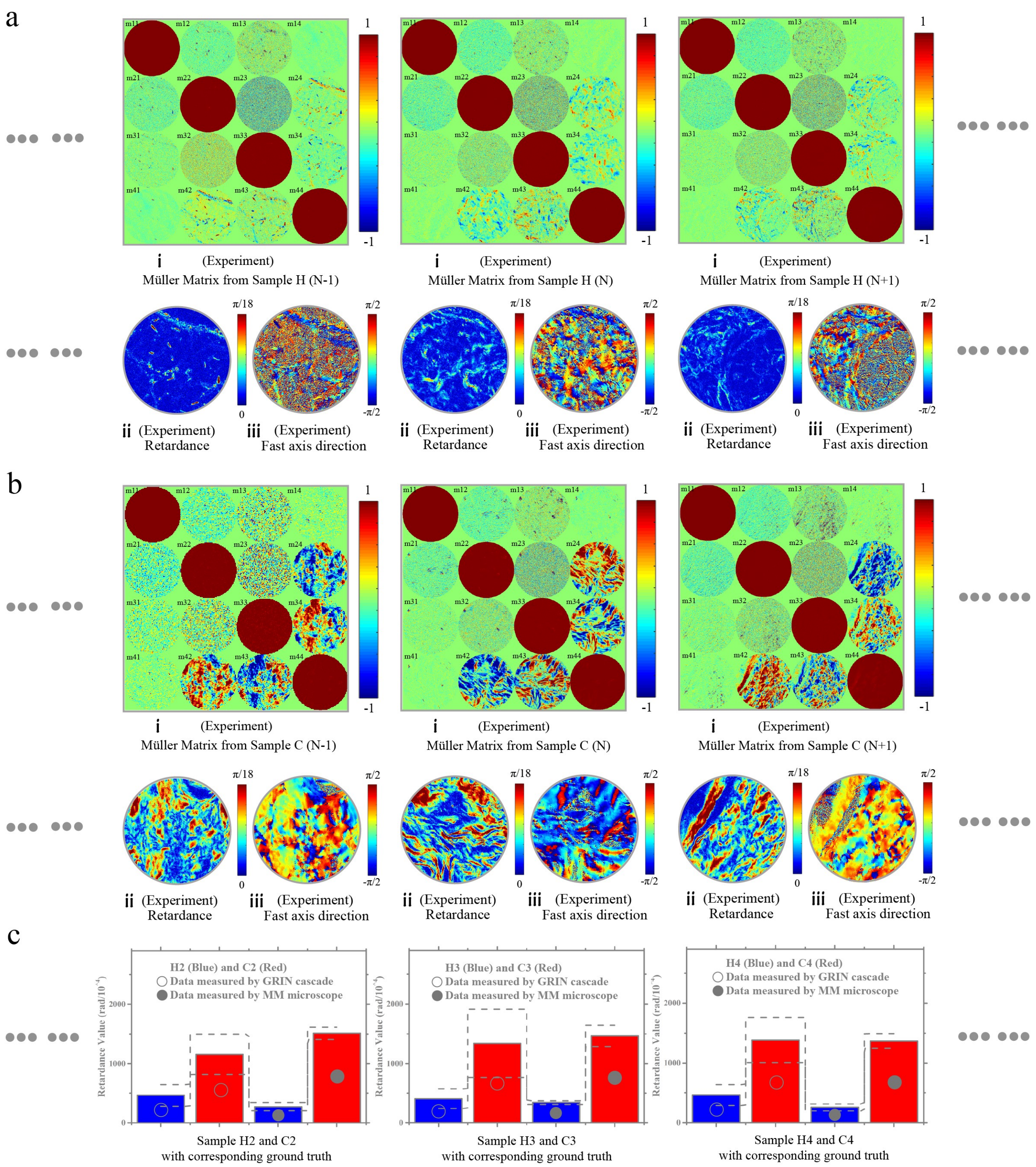

Supplementary Figure 11. Original MMs of healthy and cancerous samples. (**a**) Demonstrations of the original MMs (i), MMPD parameters (retardance (ii) and fast axis directions (iii)) of the healthy samples. (**b**) Demonstrations of the original MMs, MMPD parameters (retardance (ii) and fast axis directions (iii)) of the cancerous samples. (**c**) Demonstrations of statistic histograms (mean value and the standard deviation of the retardance) of different samples measured by the GRIN lens cascade and a conventional MM microscope (ground truth); numbers refer to Supplementary Table 1. Source data are provided as a Source Data file.

For simplicity of measurement by the MM polarimeter we make the assumption that measurement of the average SOP should be sufficient for discrimination purposes. We first defocused the GRIN lens image, in order to implement optical averaging of the polarization effects of spatially varying structures. Then we began with a chosen region with optimal CN (related to the 132° retardance[18]) to calculate the MM for demonstration of feasibility. If strong non-uniform area occurred such as near to the boundary between tissue types, we chose alternative rings (corresponding to other retardance values within the effective CN region on the PSA[52]) or applied another single-shot to reconstruct the MM. In principle, alternative region shapes can be chosen for optimization of the processes.

All the obtained data measured by the MM polarimeter were used to draw an overall 3-D dot distribution in Fig. 4f in the main article. Then, combined with the corresponding MM microscope data, Supplementary Table 1 was formed to show the data measured by two approaches as well as the corresponding P-value[53], which shows the significant difference between the two classes of samples. Considering the projection on Y (sample number) - Z (retardance) plane in the main article (Fig. 4f) and the P-values in Supplementary Table 1 of either individual samples or the overall combination, it can be seen that the polarimeter is able to distinguish efficiently the healthy and cancerous tissues. The difference between two kinds of samples is obvious across the tested samples; this also confirms the robustness of the polarimeter. Compared to the data measured by the MM microscope, larger standard deviations from the data measured by the polarimeter can be observed. The difference might result from the inhomogeneous sample structure in the effective FOV. To make the process more efficient and precise, further detailed error analysis and corresponding optimization will be the subject of further work.

Supplementary Table 1. Value of retardance δ (rad) of samples used in this section

	Slide H1 and Slide C1					Slide H2 and Slide C2				
	Healthy		***Cancerous***			***Healthy***		***Cancerous***		
	mean value	*standard deviation*	*mean value*	*standard deviation*	**P-value*	*mean value*	*standard deviation*	*mean value*	*standard deviation*	**P-value*
**GRIN cascade**	0.0365	0.0166	0.1412	0.0497	<0.001	0.0463	0.0185	0.1159	0.0338	<0.001
**MM microscope**	0.0309	0.0089	0.1289	0.0151	<0.001	0.0271	0.0067	0.1513	0.0104	<0.001
	**Slide H3 and Slide C3**					**Slide H4 and Slide C4**				
	Healthy		***Cancerous***			***Healthy***		***Cancerous***		
	mean value	*standard deviation*	*mean value*	*standard deviation*	**P-value*	*mean value*	*standard deviation*	*mean value*	*standard deviation*	**P-value*
**GRIN cascade**	0.0406	0.0164	0.1340	0.0573	<0.001	0.0464	0.0178	0.1385	0.0377	<0.001
**MM microscope**	0.0314	0.0033	0.1467	0.0179	<0.001	0.0257	0.0056	0.1371	0.0122	<0.001
	**...**					**Combination of all slides**				
	Healthy		***Cancerous***			***Healthy***		***Cancerous***		
	mean value	*standard deviation*	*mean value*	*standard deviation*	**P-value*	*mean value*	*standard deviation*	*mean value*	*standard deviation*	**P-value*
**GRIN cascade**	**...**					0.0408	0.0189	0.1324	0.0433	<0.001
**MM microscope**						0.0323	0.0102	0.1279	0.0171	<0.001

*the P-value is obtained by the significance test method[53] (*P*<=0.05 is significant), which shows the significance of the difference between the two sets of data.

Supplementary Reference

1. Born, M. & Wolf, E. Principles of optics: electromagnetic theory of propagation, interference and diffraction of light. (Elsevier, 2013).
2. Goldstein, D.H. Polarized Light, revised and expanded. (CRC press, 2003).
3. Azzam, R. Photopolarimetric measurement of the Mueller matrix by Fourier analysis of a single detected signal. *Optics Letters* **2**, 148-150 (1978).
4. Goldstein, D.H. & Chipman, R.A. Error analysis of a Mueller matrix polarimeter. *JOSA A* **7**, 693-700 (1990).
5. Smith, M.H. Optimization of a dual-rotating-retarder Mueller matrix polarimeter. *Applied Optics* **41**, 2488-2493 (2002).
6. Lu, S.-Y. & Chipman, R.A. Interpretation of Mueller matrices based on polar decomposition. *JOSA A* **13**, 1106-1113 (1996).
7. Ghosh, N. & Vitkin, A.I. Tissue polarimetry: concepts, challenges, applications, and outlook. *Journal of Biomedical Optics* **16**, 110801 (2011).

8. Sun, M. et al. Characterizing the microstructures of biological tissues using Mueller matrix and transformed polarization parameters. *Biomedical Optics Express* **5**, 4223-4234 (2014).

9. He, C. et al. Characterizing microstructures of cancerous tissues using multispectral transformed Mueller matrix polarization parameters. *Biomedical Optics Express* **6**, 2934-2945 (2015).

10. Qi, J. & Elson, D.S. A high definition Mueller polarimetric endoscope for tissue characterisation. *Scientific Reports* **6**, 25953 (2016).

11. Li, P., Lv, D., He, H. & Ma, H. Separating azimuthal orientation dependence in polarization measurements of anisotropic media. *Optics Express* **26**, 3791-3800 (2018).

12. Arteaga, O., Garcia-Caurel, E. & Ossikovski, R. Anisotropy coefficients of a Mueller matrix. *JOSA A* **28**, 548-553 (2011).

13. Samlan, C. & Viswanathan, N.K. Field-controllable Spin-Hall Effect of Light in Optical Crystals: A Conoscopic Mueller Matrix Analysis. *Scientific Reports* **8**, 2002 (2018).

14. Schulz, M. et al. Giant intrinsic circular dichroism of prolinol-derived squaraine thin films. *Nature Communications* **9**, 2413 (2018).

15. Huland, D.M. et al. In vivo imaging of unstained tissues using long gradient index lens multiphoton endoscopic systems. *Biomedical Optics Express* **3**, 1077-1085 (2012).

16. Ghatak, A. Optics. (McGraw-Hill, 2005).

17. Berry, H.G., Gabrielse, G. & Livingston, A. Measurement of the Stokes parameters of light. *Applied Optics* **16**, 3200-3205 (1977).

18. Sabatke, D. et al. Optimization of retardance for a complete Stokes polarimeter. *Optics Letters* **25**, 802-804 (2000).

19. He, C. et al. Linear polarization optimized Stokes polarimeter based on four-quadrant detector. *Applied Optics* **54**, 4458-4463 (2015).

20. Maurer, C., Jesacher, A., Fürhapter, S., Bernet, S. & Ritsch-Marte, M. Tailoring of arbitrary optical vector beams. *New Journal of Physics* **9**, 78 (2007).

21. Wang, H., Shi, L., Lukyanchuk, B., Sheppard, C. & Chong, C.T. Creation of a needle of longitudinally polarized light in vacuum using binary optics. *Nature Photonics* **2**, 501 (2008).

22. Zhan, Q. Cylindrical vector beams: from mathematical concepts to applications. *Advances in Optics and Photonics* **1**, 1-57 (2009).

23. Youngworth, K.S. & Brown, T.G. Focusing of high numerical aperture cylindrical-vector beams. *Optics Express* **7**, 77-87 (2000).

24. Devlin, R.C., Ambrosio, A., Rubin, N.A., Mueller, J.B. & Capasso, F. Arbitrary spin-to–orbital angular momentum conversion of light. *Science* **358**, 896-901 (2017).

25. Kimura, W. et al. Laser acceleration of relativistic electrons using the inverse Cherenkov effect. *Physical Review Letters* **74**, 546 (1995).

26. Niziev, V. & Nesterov, A. Influence of beam polarization on laser cutting efficiency. *Journal of Physics D: Applied Physics* **32**, 1455 (1999).

27. Beckley, A.M., Brown, T.G. & Alonso, M.A. Full poincaré beams. *Optics Express* **18**, 10777-10785 (2010).

28. Cardano, F., Karimi, E., Marrucci, L., de Lisio, C. & Santamato, E. Generation and dynamics of optical beams with polarization singularities. *Optics Express* **21**, 8815-8820 (2013).

29. Han, W., Cheng, W. & Zhan, Q. Flattop focusing with full Poincaré beams under low numerical aperture illumination. *Optics Letters* **36**, 1605-1607 (2011).

30. Camacho, J. & Tentori, D. Polarization optics of GRIN lenses. *Journal of Optics A: Pure and Applied Optics* **3**, 89 (2001).

31. Vasil'ev, V. & Soskin, M. Topological and morphological transformations of developing singular paraxial vector light fields. *Optics Communications* **281**, 5527-5540 (2008).

32. Kumar, V., Philip, G.M. & Viswanathan, N.K. Formation and morphological transformation of polarization singularities: hunting the monstar. *Journal of Optics* **15**, 044027 (2013).

33. Kumar, V. & Viswanathan, N.K. Topological structures in vector-vortex beam fields. *JOSA B* **31**, A40-A45 (2014).

34. Galvez, E.J. & Khajavi, B. Monstar disclinations in the polarization of singular optical beams. *JOSA A* **34**, 568-575 (2017).

35. Bauer, T. et al. Observation of optical polarization Möbius strips. *Science* **347**, 964-966 (2015).

36. Radwell, N., Hawley, R., Götte, J. & Franke-Arnold, S. Achromatic vector vortex beams from a glass cone. *Nature Communications* **7**, 10564 (2016).

37. Allen, L., Beijersbergen, M.W., Spreeuw, R. & Woerdman, J. Orbital angular momentum of light and the transformation of Laguerre-Gaussian laser modes. *Physical Review A* **45**, 8185 (1992).

38. Mair, A., Vaziri, A., Weihs, G. & Zeilinger, A. Entanglement of the orbital angular momentum states of photons. *Nature* **412**, 313 (2001).

39. Dennis, M., O'Holleran, K. & Padgett, M. (Elsevier, 2009).

40. Bliokh, K.Y., Rodríguez-Fortuño, F.J., Nori, F. & Zayats, A.V. Spin–orbit interactions of light. *Nature Photonics* **9**, 796 (2015).

41. Friese, M., Nieminen, T., Heckenberg, N. & Rubinsztein-Dunlop, H. Optical alignment and spinning of laser-trapped microscopic particles. *Nature* **394**, 348 (1998).

42. Garcés-Chávez, V. et al. Observation of the transfer of the local angular momentum density of a multiringed light beam to an optically trapped particle. *Physical Review Letters* **91**, 093602 (2003).

43. Padgett, M. & Bowman, R. Tweezers with a twist. *Nature Photonics* **5**, 343 (2011).

44. Ngcobo, S., Litvin, I., Burger, L. & Forbes, A. A digital laser for on-demand laser modes. *Nature Communications* **4**, 2289 (2013).

45. Wang, J. et al. Terabit free-space data transmission employing orbital angular momentum multiplexing. *Nature Photonics* **6**, 488 (2012).

46. Willner, A.E. et al. Optical communications using orbital angular momentum beams. *Advances in Optics and Photonics* **7**, 66-106 (2015).

47. Zhao, N., Li, X., Li, G. & Kahn, J.M. Capacity limits of spatially multiplexed free-space communication. *Nature Photonics* **9**, 822 (2015).

48. Yu, N. et al. Light propagation with phase discontinuities: generalized laws of reflection and refraction. *Science* **334**, 333-337 (2011).

49. Parigi, V. et al. Storage and retrieval of vector beams of light in a multiple-degree-of-freedom quantum memory. *Nature Communications* **6**, 7706 (2015).

50. Wang, X.-L. et al. Quantum teleportation of multiple degrees of freedom of a single photon. *Nature* **518**, 516 (2015).

51. Malik, M. et al. Multi-photon entanglement in high dimensions. *Nature Photonics* **10**, 248 (2016).

52. Chang, J. et al. Division of focal plane polarimeter-based 3× 4 Mueller matrix microscope: a potential tool for quick diagnosis of human carcinoma tissues. *Journal of Biomedical Optics* **21**, 056002 (2016).

53. Dong, Y. et al. Quantitatively characterizing the microstructural features of breast ductal carcinoma tissues in different progression stages by Mueller matrix microscope. *Biomedical Optics Express* **8**, 3643-3655 (2017).
